# *Fasciola hepatica* serine protease inhibitor family (serpins): Purposely crafted for regulating host proteases

**DOI:** 10.1371/journal.pntd.0008510

**Published:** 2020-08-06

**Authors:** Carolina De Marco Verissimo, Heather L. Jewhurst, Irina G. Tikhonova, Rolf T. Urbanus, Aaron G. Maule, John P. Dalton, Krystyna Cwiklinski

**Affiliations:** 1 Centre for One Health and Ryan Institute, School of Natural Sciences, National University of Ireland Galway, Galway, Ireland; 2 School of Pharmacy, Medical Biology Centre, Queen's University Belfast, Belfast, United Kingdom; 3 Thrombosis and Hemostasis Laboratory, Department of Clinical Chemistry and Hematology, University Medical Center Utrecht, Utrecht, Netherlands; 4 Microbe & Pathogen Biology, The Institute for Global Food Security, School of Biological Sciences, Queen's University Belfast, Belfast, United Kingdom; The University of Melbourne, AUSTRALIA

## Abstract

Serine protease inhibitors (serpins) regulate proteolytic events within diverse biological processes, including digestion, coagulation, inflammation and immune responses. The presence of serpins in *Fasciola hepatica* excretory-secretory products indicates that the parasite exploits these to regulate proteases encountered during its development within vertebrate hosts. Interrogation of the *F*. *hepatica* genome identified a multi-gene serpin family of seven members that has expanded by gene duplication and divergence to create an array of inhibitors with distinct specificities. We investigated the molecular properties and functions of two representatives, FhSrp1 and FhSrp2, highly expressed in the invasive newly excysted juvenile (NEJ). Consistent with marked differences in the reactive centre loop (RCL) that executes inhibitor-protease complexing, the two recombinant *F*. *hepatica* serpins displayed distinct inhibitory profiles against an array of mammalian serine proteases. In particular, rFhSrp1 efficiently inhibited kallikrein (*K*_i_ = 40 nM) whilst rFhSrp2 was a highly potent inhibitor of chymotrypsin (*K*_i_ = 0.07 nM). FhSrp1 and FhSrp2 are both expressed on the NEJ surface, predominantly around the oral and ventral suckers, suggesting that these inhibitors protect the parasites from the harmful proteolytic effects of host proteases, such as chymotrypsin, during invasion. Furthermore, the unusual inhibition of kallikrein suggests that rFhSrp1 modulates host responses such as inflammation and vascular permeability by interfering with the kallikrein-kinin system. A vaccine combination of rFhSrp1 and rFhSrp2 formulated in the adjuvant Montanide ISA 206VG elicited modest but non-significant protection against a challenge infection in a rat model, but did induce some protection against liver pathogenesis when compared to a control group and a group vaccinated with two well-studied vaccine candidates, *F*. *hepatica* cathepsin L2 and L3. This work highlights the importance of *F*. *hepatica* serpins to regulate host responses that enables parasite survival during infection and, coupled with the vaccine data, encourages future vaccine trials in ruminants.

## Author summary

Serpins are protease inhibitors that regulate various biological processes, including digestion, blood coagulation, inflammation and immune responses. The liver fluke, *Fasciola hepatica*, produces an array of inhibitors to regulate proteolytic enzymes they encounter during development within the mammalian host. In this study, we identified seven different serpins that have evolved to inhibit a range of host proteases. In particular, we characterized two representatives, FhSrp1 and FhSrp2, that we found highly expressed on the surface of the invasive newly excysted juvenile (NEJ), suggesting that they protect the parasites from harmful proteolytic effects during invasion. Contrasting inhibitory profiles were observed; while recombinant FhSrp1 inhibited kallikrein, recombinant FhSrp2 was a highly potent inhibitor of chymotrypsin. The unusual inhibition of kallikrein suggests that rFhSrp1 influences host responses such as inflammation and vascular permeability by interfering with the kallikrein-kinin system. Conversely, chymotrypsin is typically inhibited by trematode-specific serpins, implying a conserved mechanism to regulate digestive enzymes. The ability of the liver fluke serpin family to inhibit such an array of proteases highlights the importance of these inhibitors in parasite-host interactions and encourages future investigations of serpins as candidate anti-parasite vaccine targets for the control of fasciolosis in ruminants.

## Introduction

*Fasciola hepatica* is a trematode parasite that infects a wide range of mammals, most importantly humans and their domestic livestock [1]. The disease causes major economic losses to the global agricultural community [2]. Currently, ~17 million people are infected with the parasite and ~180 million are at risk of infection [3, 4]. This emerging problem prompted WHO to recently include fasciolosis amongst the most important food-borne global neglected tropical diseases (NTD) of humans [5].

Infection with *F*. *hepatica* is initiated when the mammalian host consumes vegetation contaminated with encysted metacercariae. The metacercariae excyst in the host’s intestine and the invasive newly excysted juveniles (NEJ) rapidly burrow through the gut wall and migrate to the liver. The juvenile parasite migrates through the liver parenchyma for approximately 8 to 10 weeks, feeding on the tissue to support its growth and development. During this time it must overcome physical barriers presented by the tissues and also deal with the humoral and cellular responses that the host rapidly elicits [6]. Parasites that survive unharmed and successfully reach the biliary duct develop as hermaphroditic adults and produce off-spring for decades [3]. Successful parasite infection depends, therefore, on the parasite’s ability to promptly and effectively deal with the different environments and immune responses within its host.

Many molecules produced and secreted by NEJ are well characterized and are known to aid the parasite’s tissue invasion and feeding activity [7–9]. These include several developmentally-regulated cathepsin L- and B-like cysteine proteases that are specially adapted to degrading host tissue proteins, such as collagen and fibronectin, and blood proteins such haemoglobin [10, 11]. Additionally, several cysteine protease inhibitors, such as cystatins [12] and Kunitz-type inhibitors [13] have been shown to be co-secreted with the cysteine proteases and are proposed to be important in regulating the parasite protease/anti-protease balance.

Recent proteomic studies by Di Maggio et al. [14] and Cwiklinski and Dalton [7] have shown that serine protease inhibitors (serpins) are abundant in the somatic extracts and secretomes of various life-stages of *F*. *hepatica*, although their functions remain undefined. Serpins have been described in viruses, bacteria, plants, invertebrates and vertebrates and while primarily regarded as serine protease inhibitors [15], in some cases they can also inhibit cysteine proteases [16]. Several human serpins have been shown to regulate serine proteases associated with processes such as digestion, coagulation, inflammation and immune responses [17, 18]. Helminth parasites also express serpins and may use these to target host proteases in the digestive tract or those released from neutrophil and mast cell granules [19, 20]. The trematode-specific serpins that have been functionally characterised to date predominantly exhibit inhibition of chymotrypsin-like serine proteases typically found in the digestive tract [19, 21, 22] and endogenous or pancreatic elastases [23, 24]. Beyond that, a serpin found on the surface of the head gland and spines of *Schistosoma mansoni* may regulate endogenous proteases and/or be important for intradermal and intravenous parasite survival [25].

The presence of serpins in the secretome of juvenile, immature and adult *F*. *hepatica* and the contrary lack of any serine proteases indicated to us that the serpins secreted by this parasite are adapted to regulate host proteolytic activity. This seemed a reasonable assumption given the range of proteases the parasite is likely to encounter during its migration, feeding and development within the definitive host, including chymotrypsin, trypsin, neutrophil elastases, chymase and various coagulation factors. In this study, therefore, we sought to understand the role of *F*. *hepatica* serpins in the host-parasite interaction. Following interrogation of recently acquired genomic and transcriptomic data we now describe a complete family of seven serpins within the genome of *F*. *hepatica* that are differentially transcribed throughout its lifecycle. We have functionally characterised the inhibitory profile of two recombinant representatives, FhSrp1 and FhSrp2, which are highly expressed during the invasive NEJ stage and are localised to the parasite’s surface. rFhSrp1 and rFhSrp2 are potent inhibitors of serine proteases, and exhibit greatest activity against kallikrein and chymotrypsin, respectively, with inhibitory constants in the nano-molar range. Molecular modelling studies show that the specificity between the serpins and their cognate proteases is defined by the residues found within the catalytic reactive centre loop (RCL) of the serpin, primarily in the P1-P1’ cleavage site. The diversity of RCLs in the *F*. *hepatica* serpin family, and indeed amongst serpins from a number of trematode parasites, suggests that gene duplication has created a complex variety of serpins adapted for the inhibition of a myriad of host serine and, possibly, cysteine proteases. Finally, vaccine trials in rodent models encourage the future investigation of serpins as anti-parasite vaccine candidates.

## Methods

### Ethics statement

All animal experimental procedures were carried out at Queen’s University Belfast, UK, under license from the Home Office by the Animal (Scientific Procedures) Act 1986 (License No. PPL/2806) after ethical review by the Queen’s University Animal Welfare and Ethical Review Body.

### Identification of the serpin gene family within the *F*. *hepatica* genome

Six putative serpin proteins were initially identified by proteomics of the newly excysted juvenile (NEJ) secretomes [6], based on the putative annotation of the gene models from the *F*. *hepatica* genome using *in silico* tools (Uniprot, Gene Ontology (GO) and InterproScan) [11]. Further interrogation of the *F*. *hepatica* genome using BLAST and based on putative annotation, revealed a family of serpins comprised of seven genes. Manual curation and detection of known serpin protein motifs, namely NAVYFKG and DVNEEG [26, 27] using ScanProsite (https://prosite.expasy.org/scanprosite/), and identification of the characteristic serpin RCL sequence was used to predict the full-length sequences. Confirmation of the *FhSrp7* gene sequence was also based on sequence data available in the *F*. *hepatica* genome generated by McNulty et al. [28] and the adult transcriptome data generated by Young et al. [29]. Identification of the signal peptide sequence and *N-*glycosylation sites within the serpin proteins was carried out using SignalP 4.0 Server (http://www.cbs.dtu.dk/services/SignalP-4.1/) [30] and NetNGlyc 1.0 Server (http://www.cbs.dtu.dk/services/NetNGlyc/), respectively. Gene identifiers within the *F*. *hepatica* genome (PRJEB6687) [11] and the current revised assembly (PRJEB2528) are presented within S2 Table.

### Transcriptome analysis of the *F*. *hepatica* serpin gene family

Differential gene transcription of the *F*. *hepatica* serpin genes throughout the lifecycle within the definitive mammalian host was investigated using the available *F*. *hepatica* transcriptome data (European Nucleotide Archive accession number PRJEB6904) [11], represented as the number of transcripts per million (TPM).

### Phylogenetic analysis

Homologous serpin sequences from the phylum Platyhelminthes were identified using BLAST analysis of the publicly available genome databases at WormBase ParaSite (http://parasite.wormbase.org/index.html. Version: WBPS14 (WS27)). Previously functionally characterised trematode serpins were also included in the analysis [19, 23–25, 31–33]. Protein alignments were carried out using MAFFT with the ginsi options [34], based on the protein sequence Arg^224^-Glu^374^ (FhSrp1 nomenclature) that includes the RCL region. Phylogenetic trees were constructed with PhyML 3.0 [35] using the phylogenetic model LG +G for the *F*. *hepatica*-specific tree and LG +G+I for the phylum Platyhelminthes tree, with five random starting trees and 1000 bootstrap support. The final tree figures were generated using FigTree (http://tree.bio.ed.ac.uk/software/figtree/).

### Confirmation of the *FhSrp1* and *FhSrp2* gene sequences

Total RNA was extracted from *F*. *hepatica* adult parasites using TRIzol (ThermoFisher Scientific) and the cDNA synthesized using the High-Capacity cDNA Reverse Transcription Kit (ThermoFisher Scientific), according to the manufacturer’s instructions. PCR reactions were performed in a reaction volume of 10 μL, containing 0.2 μL cDNA, 1 x DreamTaq PCR Master Mix (ThermoFisher Scientific) and 20 nM of each primer. Primers were designed to the regions encoding the start and stop codons, respectively, based on the *in silico* analysis; FhSrp1: forward primer 5′-ATGGAGAAGTCACTTTTGAAG-3′, reverse primer 5’- CGTAACATCGGCTGAAGAGTAG-3’; FhSrp2: forward primer 5′-ATGACCTCATCTATGGAACATTC-3′, reverse primer 5’- CTGATCCGGAGGTGAACTAA-3’. The PCR cycling conditions consisted of initial denaturation at 95°C for 5 min, followed by 30 cycles at 95°C for 30 sec, 50.7°C for 30 sec, 72°C for 1 min, and a final extension at 72°C for 5 min. Positive PCR products were cloned from the PCR reaction using the TOPO cloning system (ThermoFisher Scientific) and transformed into TOP10 *Escherichia coli* competent cells (ThermoFisher Scientific) according to the manufacturer’s instructions. Positive transformants were selected and the plasmid DNA purified using a PureLink HiPure Plasmid DNA Purification Kit (ThermoFisher Scientific). Plasmid DNA sequencing was performed by Source Bioscience (UK). The resulting sequences were compared with the *in silico* predicted gene sequences from the *F*. *hepatica* genome by alignment using Clustal Omega.

### Production of functional recombinant FhSrp1 and FhSrp2 serpins in *Escherichia coli* cells

The *FhSrp1* and *FhSrp2* genes were codon optimized for expression in *E*. *coli* and synthesized in the kanamycin resistant pET-28a(+) vector with a C-terminal His-tag (Genscript Biotech). The synthesized vectors were transformed into BL21 competent *E*. *coli* cells (ThermoFisher Scientific), which were grown in LB broth containing 1μg/mL kanamycin at 37°C to an OD_600._ Protein expression was induced with 1 mM isopropyl-β-D-1-thiogalactopyranoside (IPTG; ThermoFisher Scientific) for 6 hr at 24°C. Following centrifugation at 10,000 x *g* for 10 min at 4°C, the recovered bacteria pellets were treated with lysozyme (10 μg/mL), sonicated on ice and centrifuged 15,000 x *g* at 4°C for 30 min. The resulting supernatant that contained the soluble recombinant serpin was diluted 1:4 in lysis buffer (sodium phosphate buffer, pH 8, 10 mM imidazole) and passed through a column containing Ni-NTA beads (Qiagen). After washing the column with 10 mL of wash buffer (50 mM sodium phosphate buffer, pH 8, 20 mM imidazole), the recombinant protein was eluted using 5 mL of elution buffer (50 mM sodium phosphate buffer, pH 7, 250 mM imidazole). The protein purified was buffer-exchanged in 1x PBS, pH 7.4, and protein concentration and purity were verified by Bradford Protein Assay (Bio-Rad) and the proteins visualised on 4–20% SDS-PAGE gels (Bio-Rad) stained with Biosafe Coomassie (Bio-Rad). To further confirm the expression and purification of the recombinant proteins, Western blots were performed using a monoclonal mouse anti-polyhistidine antibody (1:10,000) (Sigma-Aldrich) as a primary antibody followed by incubation with a secondary antibody alkaline phosphatase conjugated goat to mouse-anti-IgG diluted 1:5,000 (Sigma-Aldrich).

### Preparation of *F*. *hepatica* NEJ and adult somatic extracts and excretory-secretory products

*F*. *hepatica* metacercariae (Italian isolate, Ridgeway Research) were excysted as previously described [9]. Briefly, the outer cyst wall was removed by agitation in 2% sodium hypochlorite solution. The metacercariae were then incubated in excystment medium (1.2% sodium bicarbonate, 0.9% sodium chloride, 0.2% sodium tauroglycocholate, 0.07% concentrated hydrochloric acid, 0.006% L-cysteine) and incubated for up to 3 hr at 37°C in 5% CO_2_. The excysted NEJ were cultured for 24 hr in RPMI-1640 medium (ThermoFisher Scientific) containing 2 mM L-glutamine, 30 mM HEPES, 0.1% (*w/v*) glucose, and 2.5 μg/mL gentamycin with or without 10% foetal calf serum at 37°C with 5% CO_2_ followed by centrifugation at 400 x *g* to recover the excretory-secretory (ES) protein fraction. The resulting ES was concentrated using Amicon Ultra 3kDa columns (Merck Millipore) [6].

NEJ and adult somatic proteins were extracted by homogenisation in RIPA buffer (Sigma-Aldrich) containing protease and phosphatase inhibitor cocktails (Sigma-Aldrich). The extracted proteins were then centrifuged at 10,000 x *g* for 40 min at 4°C to remove any insoluble components. Protein concentration was measured by the Bradford Protein Assay (Bio-Rad).

### Western blot analysis of recombinant FhSrp1 and FhSrp2 and native serpins in *F*. *hepatica* NEJ and adult extracts

The rFhSrp1 (0.1 μg/lane), rFhSrp2 (0.1 μg/lane), rFhCL3 (0.1 μg/lane), somatic extract from NEJ 24 hr (30 μg/lane) and somatic extract from adult flukes (30 μg/lane) were resolved by gel electrophoresis using 4–20% precast SDS-PAGE gels (Bio-Rad) and electro-transferred onto nitrocellulose membrane, prior to incubation in blocking buffer (5% milk in PBS-0.05% Tween-20 (*v/v*)). The membranes were then probed for 1 hr at room temperature with the anti-FhSrp1, anti-FhSrp2 or anti-FhCL3 antibodies (all prepared in rabbit against recombinant FhSrp1, FhSrp2 or FhCL3, respectively; Eurogentec) at a dilution of 1:500. The membranes were washed five times and then incubated with the secondary antibody alkaline phosphatase conjugated goat anti-rabbit IgG diluted 1:10,000. The immune-reactive bands were visualised using the substrate SigmaFast BCIP/NBT (Sigma-Aldrich).

### Immunolocalization of serpins in NEJ by confocal microscopy

*F*. *hepatica* metacercariae were excysted as described above and NEJ cultured for 24 hr in RPMI 1640 medium containing 2 mM L-glutamine, 30 mM HEPES, 0.1% (*w/v*) glucose, 2.5 μg/ml gentamycin and 10% foetal calf serum (ThermoFisher Scientific). Following parasite culture the parasites were fixed in 4% paraformaldehyde (PFA) in PBS (Sigma-Aldrich), pH 7.4, for 1 hr at room temperature (RT). After three washes in antibody diluent (AbD buffer: PBS containing 0.1% (*v/v*) Triton X-100, 0.1% (*w/v*) bovine serum albumin and 0.1% (*w/v*) sodium azide), the NEJ were incubated in AbD containing anti-rFhSrp1, anti-rFhSrp2 or anti-rFhCL3 (used as non-related control) at a 1:500 dilution, overnight at 4ºC, followed by four washes in AbD. As a negative control, separate samples were incubated in AbD containing rabbit pre-immune at a 1:500 dilution. After washing, the NEJ samples were incubated with the secondary antibody, fluorescein isothiocyanate (FITC)-labelled goat anti-rabbit IgG (Sigma-Aldrich) (1:200) overnight at 4°C in the dark. To counter-stain muscle tissue, the samples were incubated in AbD containing phalloidin-tetramethylrhodamine isothiocyanate (TRITC) (200 μg/mL) overnight in the dark at 4°C. The specimens were mounted on slides using 10% glycerol solution containing 0.1 M propyl gallate. The slides were examined using an Olympus Fluoview 3000 Laser Scanning Confocal Microscope under the PL APO CS 60x oil objective lens. Olympus type F immersion oil was used in viewing and all images were taken at room temperature.

### Analysis of the rFhSrp1 and rFhSrp2 protease inhibitory activity

A broad panel of serine and cysteine proteases was used to determine the inhibitory specificity of rFhSrp1 and rFhSrp2. Enzymes, reaction conditions and substrates used for measuring protease activity are presented in Table 1. Unless highlighted, all the screening assays were performed at 37°C, in a 200 μL reaction volume of an appropriate buffer. Initially, the reaction buffer was mixed with the rFhSrp proteins (10 nM and 500 nM) and incubated for 15 min at 37°C. The protease target was then added to the reaction and incubated for a further 10 min at 37°C before the fluorogenic substrate was added. The proteolytic activity was measured over a 1 hr period, at 37°C as relative fluorescent units (RFU) in a PolarStar Omega Spectrophotometer (BMG LabTech). All assays were carried out in triplicate. Pefabloc SC (2 mM; Sigma-Aldrich) or E-64 (100 μM; Sigma-Aldrich) were used as positive control inhibitors of serine and cysteine proteases, respectively.

**Table 1:** Assay conditions for each protease activity screened with rFhSrp1 and rFhSrp2.

Enzyme	Running buffer	Substrate
Bovine α-Chymotrypsin (4 nM)	Sodium acetate^1^	Suc-Ala-Ala-Pro-Phe-NHMec (20 μM)
Bovine Trypsin (168 nM)	Sodium acetate	Z-Leu-Arg-NHMec (20 μM)
Human Chymase (0.6 μM)	Tris buffer^2^	Suc-Ala-Ala-Pro-Phe-NHMec (20 μM)
Human Neutrophil elastase (50 nM)	Hepes buffer^3^	Z-Ala-Ala-Pro-Val-NHMec (20 μM)
Human Cathepsin G (100 nM)	Sodium acetate	Suc-Ala-Ala-Pro-Phe-NHMec (100 μM)
Porcine Kallikrein (150 nM)	Sodium acetate	Z-Gly-Pro-Arg-NHMec (20 μM)
FXIIa (7.6 nM)	Tris buffer	Z-Ile-Glu-Gly-Arg-NHMec (20 μM)
FXIa (3.68 nM)	Tris buffer	Z-Gly-Pro-Arg-NHMec (20 μM)
FXa (5.8 nM)	Tris buffer	Z-Ile-Glu-Gly-Arg-NHMec (20 μM)
Bovine Thrombin (800 pM)	Sodium acetate	Z-Gly-Pro-Arg-NHMec (20 μM)
Activated protein C (0.05 μM)	Tris-PEG buffer^4^	Z-Gly-Pro-Arg-NHMec (20 μM)
Plasmin (800 μM)	Sodium acetate	Z-Leu-Arg-NHMec (20 μM)
*F*. *hepatica* cathepsin L1 (2.7 nM)	Sodium acetate	Z-Leu-Arg-NHMec (20 μM)
*F*. *hepatica* cathepsin L2 (5 nM)	Sodium acetate	Z-Leu-Arg-NHMec (20 μM)
*F*. *hepatica* cathepsin L3 (5 nM)	Sodium acetate	Z-Gly-Pro-Arg-NHMec (20 μM)
*F*. *hepatica* cathepsin B1 (135 nM)	Sodium acetate	Z-Phe-Arg-NHMec (20 μM)
*F*. *hepatica* cathepsin B2 (270 nM)	Sodium acetate	Z-Val-Iso-Arg-NHMec (20 μM)
*F*. *hepatica* cathepsin B3 (135 nM)	Sodium acetate	Z-Val-Iso-Arg-NHMec (20 μM)
Human cathepsin L (0.2 nM)	Sodium acetate	Z-Phe-Arg-NHMec (20 μM)
Human cathepsin K (2 nM)	Sodium acetate	Z-Phe-Arg-NHMec (20 μM)

1–100 nM Sodium acetate, 1 mM EDTA, 1 mM DTT, 0.01% Brij L23, pH 7.0

2–20 mM TRIS, 150 mM NaCl, 0.1% BSA, pH 7.4

3–100 mM Hepes, 500 nM NaCl, 0.00% Brij L23, pH 7.0

4–20 mM TRIS, 100 mM NaCl, 0.1% BSA, 0.1% PEG, 2.5 mM CaCl2, pH 7.4

All the *F*. *hepatica* recombinant cathepsin L and B proteases used in this study were recombinantly produced in our laboratory. Human cathepsin K, α-chymotrypsin, trypsin, kallikrein, thrombin, plasmin and chymase were acquired from Sigma-Aldrich; Human Neutrophil elastase and Cathepsin G are from Elastin Products Company; Human cathepsin L is from Enzo Life Sciences; Factors Xa, XIa and XIIa are from Molecular Innovations.

The importance of glycosaminoglycans (GAGs) for serpin inhibitory activity was tested by adding heparan sulfate (5 ng/mL; Amsbio), low molecular weight heparin (10 ng/mL; Amsbio), dermatan (10 ng/mL; Amsbio) or dextran sulfate (10 ng/mL; Sigma-Aldrich) to the reaction buffer [36].

The cathepsin cysteine protease activity in the ES proteins from NEJ 24 hr post-excystment and adult flukes was assessed in sodium acetate buffer (100 mM sodium acetate, 1 mM EDTA, 1 mM DTT, 0.01% Brij L23, pH 7.0) using the substrates Z-Gly-Pro-Arg-NHMec (20 μM) or Z-Leu-Arg-NHMec (20 μM), respectively.

The inhibition constant (*K*_i_) of the kallikrein-rFhSrp1 and chymotrypsin-rFhSrp2 complexes were determined by decreasing the concentration (nM) of rFhSrp1 or rFhSrp2 by serial dilution in assays performed under the same conditions as described above. Kallikrein activity (15 nM) was assayed with the fluorogenic substrate Gly-Pro-Arg-NHMec (9.1 μM) while chymotrypsin activity (2 μg/mL) was assayed using Ala-Ala-Pro-Phe-NHMec (42.9 μM) [37]. The estimated *K*_i_ value was determined using non-linear regression analysis in GraphPad Prism 5.0 Software (http://www.graphpad.com). Initial velocities were fitted to Morrison’s equation (Eq 1) and the resulting apparent *K*_i_ (*K*_i_ app) fitted to Eq 2 to determine the *K*_i_ of the protease in the presence of the inhibitor [38]. All the assays were carried out in triplicate.

$$\frac{vi}{vo}=1-\frac{([E]+[I]+Ki^{app}-\sqrt{{\left([E]+[I]+ki^{app}\right)}^{2}-4[E][I]}}{2[E]}$$ Eq 1

$$Ki^{app}=Ki\left(1+\frac{Km}{\left[S\right]}\right)$$ Eq 2

### Homology modelling

The homology models of FhSpr1 and FhSpr2 were built based on *Manduca sexta* serpin 1B (PDB code: 1K9O), whereas the structures of kallikrein and chymotrypsin were rebuilt from the corresponding crystal structures of these enzymes (PDB codes 1CBW and 5TJX, respectively). The homology models were built and optimized using the default homology modelling protocol of the Prime module of the Schrodinger software [39]. The protein-protein complexes were obtained via the protein structure alignment to the serpin-trypsin complex (PDB code: 1K9O), which is followed by the default energy optimization procedure of the MacroModel Dynamics and Minimization modules if the Schrodinger software. The images were generated in Maestro 12.1.013 [39].

### Coagulation assays

Calibrated automated thrombography (CAT) was used to assess the interaction of the *F*. *hepatica* serpins with coagulation factors II, V, VII, VIII, IX, X and XI as per the protocol used by Huskens et al. [40]. Thrombin generation was tested in triplicate in the presence or absence of rFhSrp1 and rFhSrp2, which were added in varying concentrations (12.5 to 50 μg/mL).

The inhibitory potential of the *F*. *hepatica* serpins against the intrinsic coagulation pathway was investigated using the activated partial thromboplastin time (APTT) test. The tests were performed in triplicate using varying concentrations of the *F*. *hepatica* serpins (12.5 to 50 μg/mL), at 37.2°C using the MC-10 coagulometer (Merlin Medical, Tredegar, UK). Plasma, with or without the recombinant *F*. *hepatica* serpins, was pre-incubated at 37.2°C, followed by the addition of 50 μL of undiluted Dade Actin FS (Siemens Healthcare Diagnostics). After 2 min incubation, 50 μL of 25 mM CaCl_2_ was added and the clotting time measured.

### SDS-stability of serpin and target protease complex

The ability of rFhSrp1-kallikrein or rFhSrp2-α-chymotrypsin to form a SDS-stable complex was assessed using gel electrophoresis under non-reducing conditions (4–20% SDS-PAGE gels (Bio-Rad). The serpin and its target protease were pre-incubated alone or together in molar ratios of 1:1, 2:1 and 3:1 in sodium acetate buffer, pH 7.0 at 37°C, for 30 min. The samples were then mixed 1:1 (*v/v*) in SDS-PAGE loading buffer and incubated for 5 min, at 95°C, resolved in 4–20% gradient gels and stained with Biosafe Coomassie (Bio-Rad). Complex formation was visualized and imaged using a G:Box Chemi XRQ imager (Syngene).

### Assessment of *F*. *hepatica* serpins as candidate vaccine targets

Seven weeks-old male rats (Sprague–Dawley) were used in two independent vaccine trials to assess the serpin proteins as candidate vaccine targets for control of fasciolosis.

**Fig 1:**
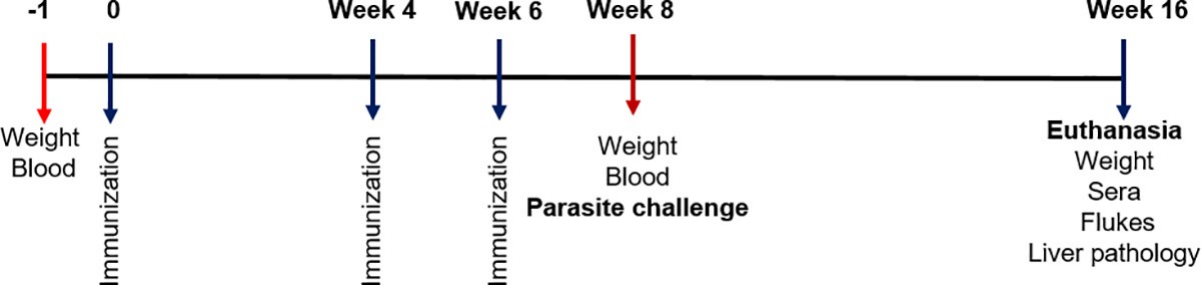
Graphical schematic showing the schedule for the *F*. *hepatica* serpin vaccine trial in rats. Red arrows show when the blood samples were taken.

The *F*. *hepatica* cathepsin L2 and L3, FhCL2 and FhCL3, are expressed by immature and newly excysted juvenile *F*. *hepatica*, respectively [11] and were produced and purified in our laboratory as recombinant proteins in yeast as previously described by Robinson et al. [41]. Vaccine trial 1 consisted of two groups: Group 1, adjuvant control group (Montanide ISA 206VG, Seppic) (n = 8); and Group 2, rFhSrp1/rFhSrp2 (rFhSrps) vaccine (n = 13) consisting of a mix of rFhSrp1 (50 μg) and rFhSrp2 (50 μg) formulated in the Montanide adjuvant (1.1 *v*/*v*). Vaccine trial 2 consisted of three groups: Group 1, adjuvant control group (Montanide ISA 206VG, Seppic) (n = 10); Group 2, rFhSrps vaccine (n = 14) consisting of a mix of rFhSrp1 (50 μg) and rFhSrp2 (50 μg) formulated in the Montanide adjuvant (1:1 *v*/*v*); and Group 3, a mix of recombinant *F*. *hepatica* cathepsin L2 (50 μg) and *F*. *hepatica* cathepsin L3 (50 μg) (rFhCLs) (n = 14) formulated in the Montanide adjuvant (1:1 *v*/*v*). Both trials consisted of a pre-evaluation of the animals on day -1 (bleeding to obtain sera samples and assessment of weight), followed by three vaccinations by subcutaneous injection in the scruff of the neck on day 0, week 4 and week 6 (Fig 1).

At week 8, the animals were orally infected with 20 *F*. *hepatica* metacercariae (Italian isolate, Ridgeway Research) administered in 70 μL water using a pipette. Eight weeks after challenge the rats were euthanized by depletion of O_2_ using CO_2_. Individual serum samples were collected and stored at -80ºC until use. The livers were recovered and the level of pathology assessed by visual assessment and by detection of liver enzymes within the sera samples (see below). The number of adult flukes in the bile ducts was enumerated.

### Evaluation of the rat immune response by ELISA

Flat-bottom 96 well microtitre plates (Nunc MaxiSorp, Biolegend) were coated with a mixture of rFhSrp1 (0.5 μg/mL) and rFhSrp2 (0.5 μg/mL) or rFhCL1 (1 μg/mL) and incubated overnight at 4ºC. After incubation in blocking buffer (5% milk in PBS-0.05% Tween-20 (*v/v*), PBST, pH 7.4) and washing steps, rat sera collected at day -1 and week 8 and 16, diluted 1:500 in blocking buffer, were added to antigen-coated wells and incubated for 1 hr at RT. After washing five times with PBST, the secondary antibody HRP goat to rat-anti-IgG (ThermoFisher Scientific) was added (1:1,000), and the plates incubated for 1 hr at RT. After washing five times, TMB substrate (3,3′,5,5′-Tetramethylbenzidine Liquid Substrate Supersensitive, Sigma-Aldrich) was added to each well. Following a five-minute incubation the reaction was stopped with 2 N sulphuric acid and plates read at 450 nm in a PolarStar Omega Spectrophotometer. All samples were analysed in triplicates. Isotype specific immune responses were assessed with monoclonal mouse to rat-anti-IgG1 or IgG2 antibodies (1:2,000) (BioLegend) as secondary antibody, followed by incubation with HRP goat to mouse anti-IgG antibody (1:5,000) (Novex).

### Assessment of liver pathology

The livers were visually inspected for gross pathology and the level of liver pathology assessed according to a score of 0 to 5 (S1 Table). Levels of the liver enzyme gamma-glutamyl transferase (GGT) were measured in rat serum samples using the GGT colorimetric kit (Abcam) using a PolarStar Omega Spectrophotometer. The GGT values were corrected for those samples that presented significant haemolysis according to the manufacturer’s instructions. Periostin levels were determined using the Rat-Periostin ELISA kit according to the manufacturer’s instructions (Abbexa). Rat serum samples (1:100) were analysed in duplicate and measured using a PolarStar Omega Spectrophotometer. The average periostin value (pg/mL) was calculated for each group.

### Statistical analysis

Statistical analysis was carried out using GraphPad Prism version 5. Differences between the animal groups were assessed using the Mann-Whitney U test or One-Way ANOVA using the Kruskal-Wallis test and 95% confidence intervals. Correlation analyses used the Spearman nonparametric correlation test with 95% confidence intervals.

## Results

### *F*. *hepatica* genome encodes a family of serpins

Interrogation of the *F*. *hepatica* genome identified seven serpin sequences, referred to herein as *FhSrp1* to *FhSrp7*. These sequences included those previously identified within the NEJ secretome [6, 14]. The classification of these sequences as serpin inhibitors was based on the presence of serpin-specific motifs NAVYFKG and DVNEEG, and the identification of the conserved RCL from comparative analysis with known serpin sequences. All seven *F*. *hepatica* serpin genes are present in both assemblies of the *F*. *hepatica* genome (PRJEB6687 and PRJEB25283); the gene identifiers from both assemblies are reported in S2 Table.

**Fig 2:**
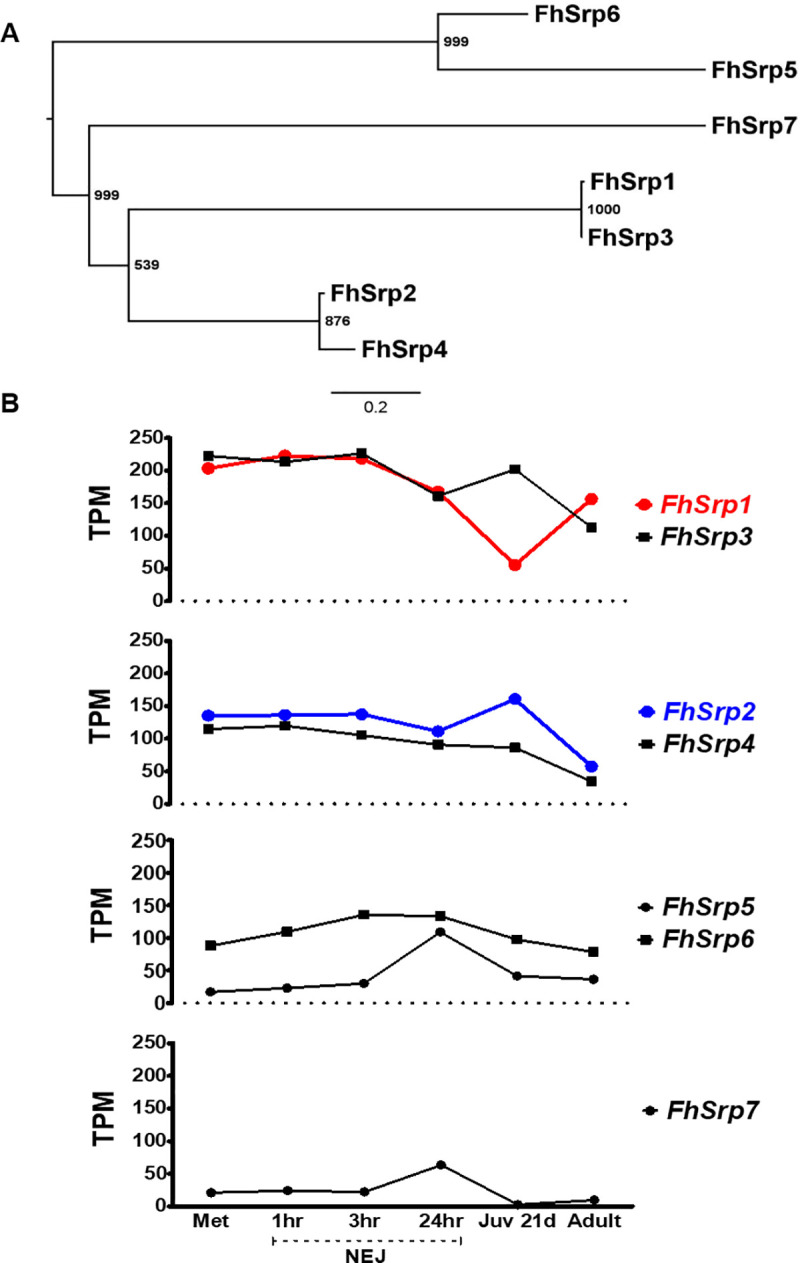
Phylogenetic analysis and gene transcription of the *Fasciola hepatica* serpin family. (A) Midpoint-rooted maximum likelihood phylogram generated by PhyML based on the protein sequence Arg^224^-Glu^374^ (FhSrp1 nomenclature) that includes the reactive centre loop (RCL) region. Bootstrap support values (1000 iterations) are shown at each node. (B) Graphical schematic of the relative gene expression for *F*. *hepatica* serpin family represented by transcripts per million (TPM) during the parasite’s lifecycle within the mammalian host: the infective metacercariae (Met), newly excysted juveniles (NEJ) at 1 hr, 3 hr and 24 hr post-excystment, liver stage juvenile parasites 21-days post infection (Juv 21d) and bile duct stage mature adult parasites (Adult). The serpin genes, *FhSrp1* and *FhSrp2* chosen for further characterisation are highlighted in red and blue, respectively.

Phylogenetic analysis based on the predicted protein sequences highlights the differences between the seven sequences, which separate into three clusters each containing two serpins (FhSrp1 and FhSrp3; FhSrp2 and FhSrp4; FhSrp5 and FhSrp6; Fig 2A). The FhSrp7 protein sequence is very different from the other *F*. *hepatica* serpin sequences and is situated on a separate branch within the phylogenetic tree. In particular, analysis of the RCL sequence within the translated protein sequences shows that all seven serpins contain the residues associated with the serpin shutter region (PROSITE signature PS00284; S1 Fig). With the exception of FhSrp7, the conserved residues within the serpin hinge region (P17-P8) are also present. However, despite these similarities, the central region of the RCL that contains the P1 active site residue is highly variable (18.5–96.7% amino acid similarity). This RCL variability suggests that the *F*. *hepatica* family of serpins is functionally divergent.

Interrogation of the available phylum Platyhelminthes genomes revealed a number of homologous serpin sequences from 17 helminth species of the Cestoda, Trematoda and Rhaditophora classes. These sequences exemplify serpins from a free-living flatworm and a range of parasitic flatworms and tapeworms capable of infecting a variety of hosts. Comparable with our analysis of the *F*. *hepatica* serpins, these sequences display high levels of sequence diversity (S2 Fig). Phylogenetic analysis separates the platyhelminth serpins into three main groups, A, B and C (with smaller clusters within these groups), with four sequences not clustering with the remaining sequences (S2 Fig). Consistent with the phylum Platyhelminthes phylogeny, the free-living flatworm, *Macrostomum lignano*, does not cluster with its parasitic counterparts. In contrast to groups A and B that do not separate by species, group C is comprised solely of sequences from the *Schistosoma* parasites. The diversity of the serpins is also evident within the RCL sequence, all of which show conservation within the hinge and shutter regions, but high levels of variability within the active site region (P4-P4’) (S2 Fig).

The *F*. *hepatica* serpins are dispersed within two of the main groups of the phylogenetic tree (groups A and B). Four of the serpins (FhSrp1 to FhSrp4) cluster with homologous sequences from the intestinal trematode *Echinostoma caproni* (Group B). In comparison, FhSrp5, FhSrp6 and FhSrp7 separate broadly with sequences from other bile dwelling flukes (Group A; S2 Fig). Surprisingly, three sequences from the cestode parasites, *Dibothriocephalus latus* (DILT_0001377201), *Hymenolepis microstoma* (HmN_003003780) and *Spirometra erinaceieuropaei* (SPER_0003298501) show 100% amino acid similarity to FhSrp2 (S3 Fig).

### The *F*. *hepatica* serpin family are temporally regulated

Our previous analysis of the *F*. *hepatica* lifecycle stage-specific transcriptomes has revealed that this parasite expresses many multi-gene families and highly regulates the transcription of the family members [6, 11]. Based on these transcriptome data we show that *F*. *hepatica* also tightly regulates the transcription of the serpin genes, which are differentially expressed throughout the lifecycle within the mammalian host. This strict control of serpin transcription likely reflects the different roles these serpins play at different points in the growth and development of the parasites in the mammalian host.

With the exception of *FhSrp5* and *FhSrp7*, the remaining five genes exhibit transcription within the NEJ stages, with the *FhSrp1* and *FhSrp3* showing the highest levels of transcription during these early infective stages (Fig 2B). These data mirror the protein expression of these serpins observed within the NEJ secretome [6]. Once the parasite is in the liver of the mammalian host, increased transcription of *FhSrp2* occurs in contrast to a decrease in *FhSrp1* expression. This pattern of expression is reversed within the adult stages with higher levels of *FhSrp1* compared with *FhSrp2*. *FhSrp7* shows the lowest level of transcription across the lifecycle of the serpin family (Fig 2B).

### Bioinformatic characterisation of FhSrp1 and FhSrp2

To investigate the function of *F*. *hepatica* serpins we selected two of the most abundantly transcribed genes, namely *FhSrp1* and *FhSrp2*. These represent members of the two main groups of *F*. *hepatica* serpins, which are highly secreted by the NEJ stage and also show contrasting developmental transcription (Fig 2) and variable residues within the putative P1 site implying different serine protease specificity (S1 Fig). Prior to recombinant expression, the *FhSrp1* and *FhSrp2* genes were amplified to confirm the *in silico* predicted gene sequence, specifically relating to the RCL encoding region. The resulting *FhSrp1* and *FhSrp2* gene sequences contain an open reading frame of 1122 and 1131 base pairs, respectively, and encode proteins of 374 and 377 residues, with predicted molecular weights of 41.3 and 41.6 kDa.

**Fig 3:**
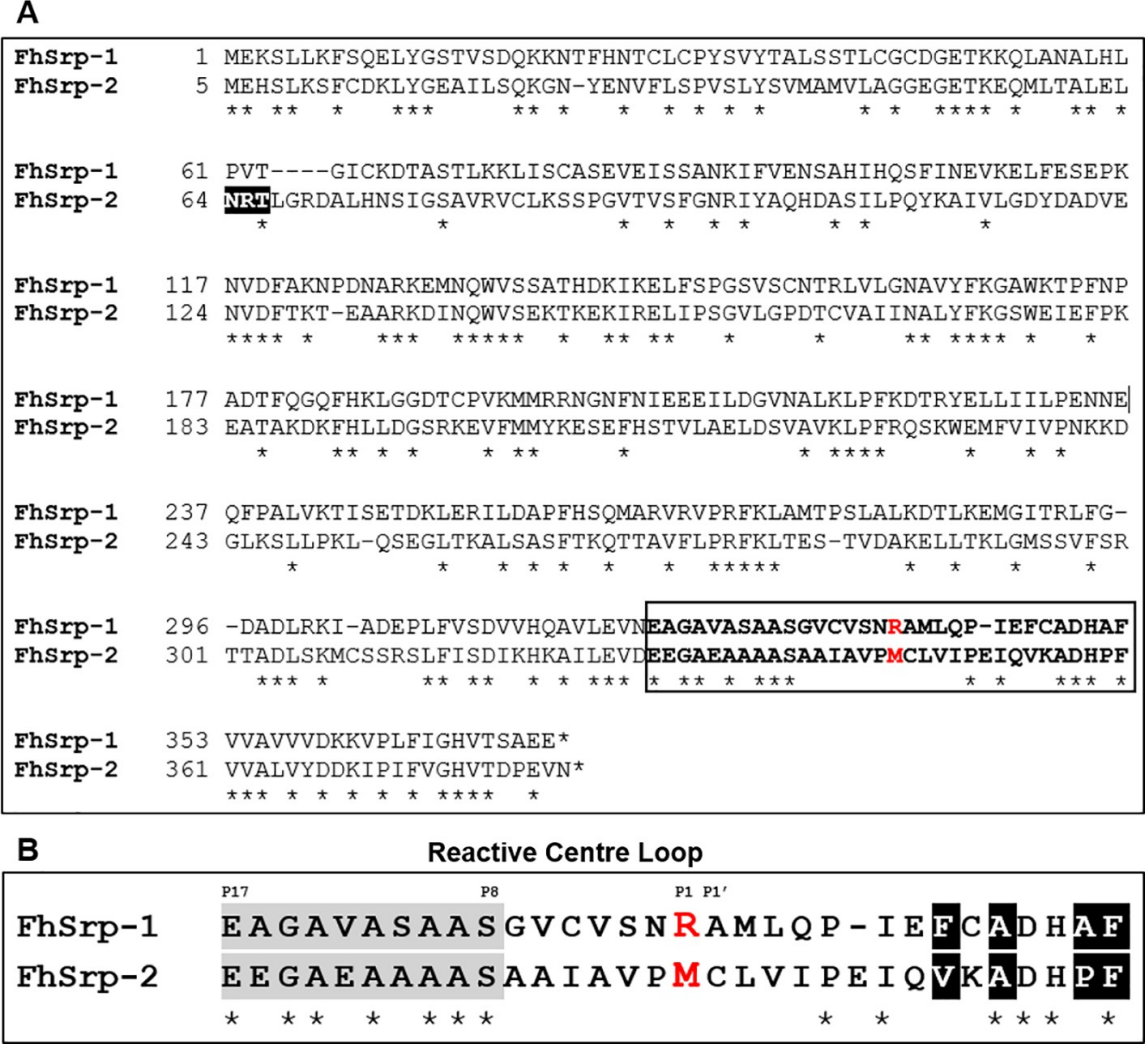
Comparative analysis of *F*. *hepatica* serpin 1 and 2 proteins. (A) Clustal Omega alignment of the FhSrp1 and FhSrp2 protein sequences. The reactive centre loop region (RCL) is boxed and the predicted P1 residue is highlighted in red. The putative *N-*glycosylation site is highlighted in black. (B) Alignment of the RCL sequences. The residues within the conserved hinge region, P17—P8, are shaded in grey, the predicted P1 position is in red and the amino acids predicted to form the shutter region (PROSITE signature PS00284) are highlighted in black. Asterisks below the alignment indicate residues identical in the two serpins.

Protein sequence analysis revealed that the sequences were complete and contained the three conserved serpin motifs, namely a hinge region (P17-P9), a signature shutter region and an RCL region (Fig 3). Both sequences did not contain an N-terminal signal peptide, indicating that alternative methods of secretion are used by these proteins. Only FhSrp2 was predicted to have one potential *N*-glycosylation site (Asp^64^), which is not located within the RCL region (Fig 3A). Comparative analysis of the two serpin proteins shows that they share 57% amino acid similarity. Importantly, the two proteins differ in the amino acids that form the active site scissile bond (P1-P1’ position), namely arginine and alanine residues in FhSrp1, compared with methionine and cysteine residues in FhSrp2 (Fig 3B). These differences forecasted that the two serpins would have distinct inhibitory specificities.

### Expression of native *F*. *hepatica* serpins

**Fig 4:**
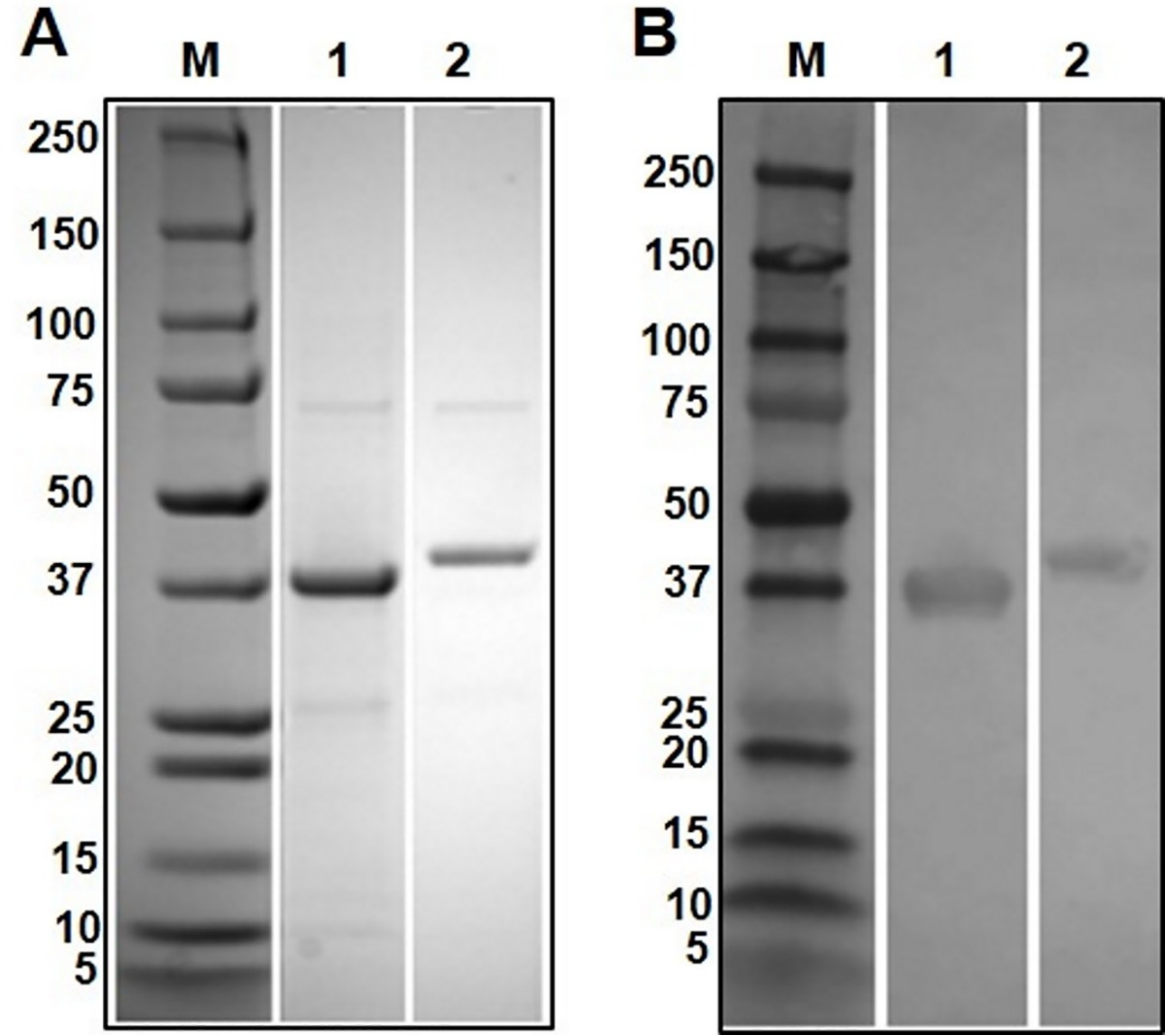
Recombinant expression of *F*. *hepatica* serpins. (A) rFhSrp1 (lane 1) and rFhSrp2 (lane 2) expressed in *E*. *coli* BL21 cells, purified by affinity column, resolved in a 4–20% SDS-PAGE gel and stained with Biosafe Coomassie; (B) rFhSrp1 (lane 1) and rFhSrp2 (lane 2) at 1 μg/lane were electro transferred onto a nitrocellulose membrane and probed with the mouse monoclonal antibody to poly-histidine (1:10.000). M: molecular weight in kDa (Precision Plus Protein Dual, Bio-Rad).

**Fig 5:**
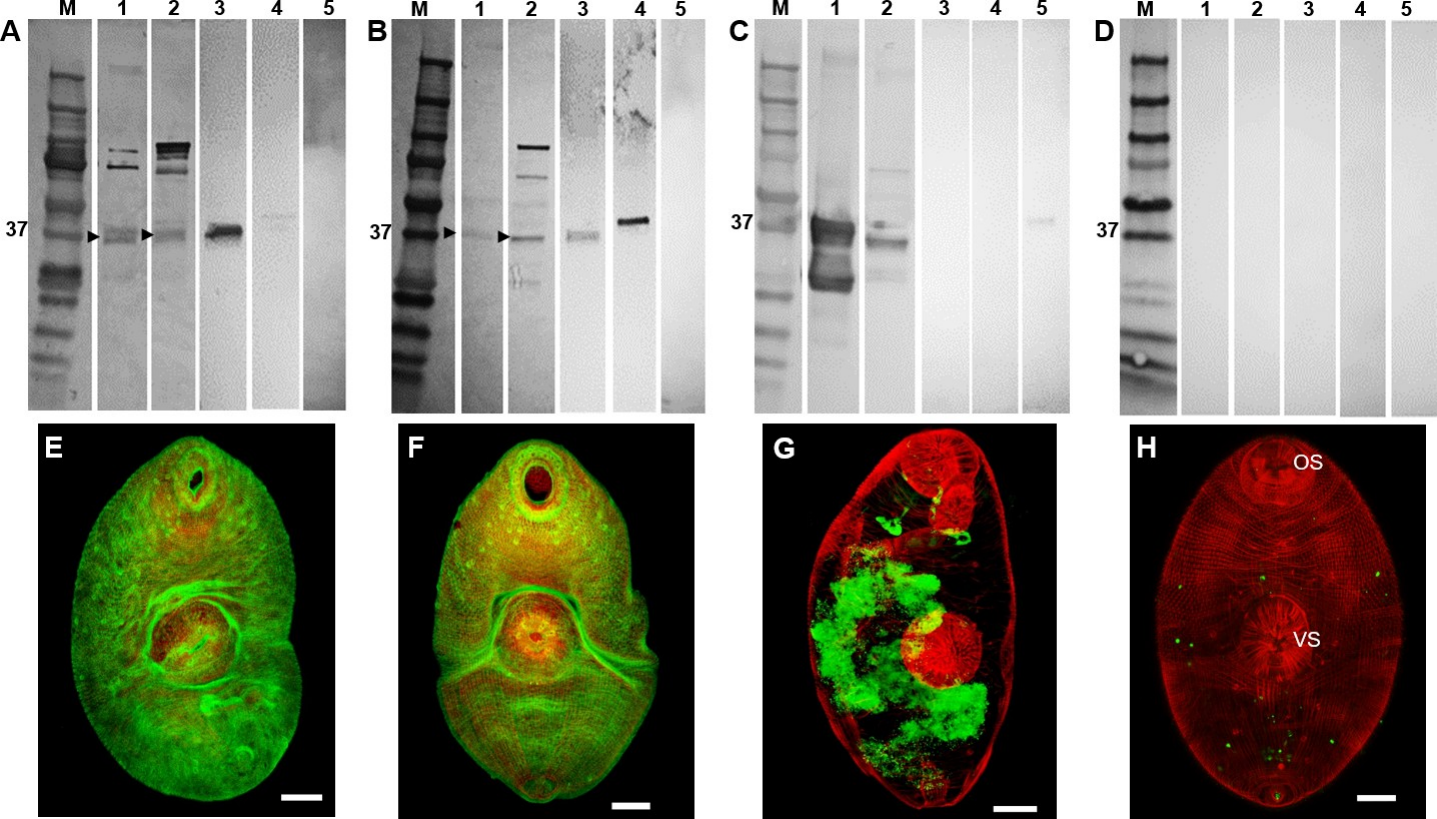
Immune detection of native *F*. *hepatica s*erpins. (A-D) Western blot analysis of somatic extracts of *F*. *hepatica* NEJ 24 hr post-excystment and adult parasites and the recombinant *F*. *hepatica* serpins FhSrp1 and FhSrp2. Lane 1: NEJ 24 hr somatic extract (30 μg/lane); lane 2: adult worm somatic extract (30 μg/lane); lane 3: recombinant rFhSrp1 (0.1 μg/lane); lane 4: recombinant rFhSrp2 (0.1 μg/lane); lane 5: recombinant rFhCL3 (0.1 μg/lane). Immunoblots were probed with (A) anti-rFhSrp1 polyclonal antibodies raised in rabbit, (B) anti-rFhSrp2 polyclonal antibodies raised in rabbit, (C) anti-rFhCL3 polyclonal antibodies raised in rabbit or (D) rabbit pre-immune serum as a negative control. M: molecular weight. (E-H) Whole mount NEJ 24 hr post-excystment immunolocalisation of the *F*. *hepatica* serpins by confocal laser microscopy represented by green fluorescence (FITC staining). (E) Fixed NEJ probed with anti-rFhSrp1 polyclonal antibodies raised in rabbit. (F) Fixed NEJ probed with anti-rFhSrp2 polyclonal antibodies raised in rabbit. (G) Fixed NEJ probed with anti-rFhCL3 polyclonal antibodies raised in rabbit. (H) Fixed NEJ probed with rabbit pre-immune anti-serum as a negative control. All samples were counter-stained with phalloidin-TRITC to stain muscle tissue (red fluorescence). OS, oral sucker. VS, ventral sucker. Scale bars, 20 μM.

Western blot analysis using antibodies raised in rabbits to the recombinantly-expressed serpins, rFhSrp1 and rFhSrp2 (Fig 4A and 4B), was carried out using the somatic extracts of the NEJ and adult fluke parasites. Each antibody identified a ~40 kDa band in both extracts, consistent with the expected molecular size for both proteins (Fig 5A and 5B, lanes 1 and 2). However, the anti-rFhSrp1 and anti-rFhSrp2 antibodies also recognised several additional bands at higher molecular weights (~50, ~70, ~100 kDa) which were not detected with an antibody prepared to the recombinant *F*. *hepatica* cathepsin L3 cysteine protease (rFhCL3) (Fig 5C, lanes 1 and 2). This result indicates that the additional higher molecular weight bands recognised by the anti-rFhSrp1 and anti-rFhSrp2 antibodies are not a result of non-specific binding. It is more likely that the higher bands represent native *F*. *hepatica* serpins complexed with other proteins or with themselves.

To investigate the location of FhSrp1 and FhSrp2 within the NEJ, we used whole mount immunolocalisation of the NEJ 24 hr post-excystment. Diffuse fluorescence was observed on the surface of the NEJ, with a brighter signal present around the oral and ventral sucker that indicates a higher concentration of these inhibitors in these regions (Fig 5E and 5F). This fluorescent staining is in contrast to that detected using antibodies against rFhCL3, which localises to the bifurcated NEJ gut, consistent with the fact that cathepsins are synthesized by the gastrodermal cells (Fig 5G). No fluorescent signal was detected by probing the NEJ with pre-immune sera (Fig 5H).

### Recombinant FhSrp1 and FhSrp2 exhibit distinct inhibitory profiles

**Fig 6:**
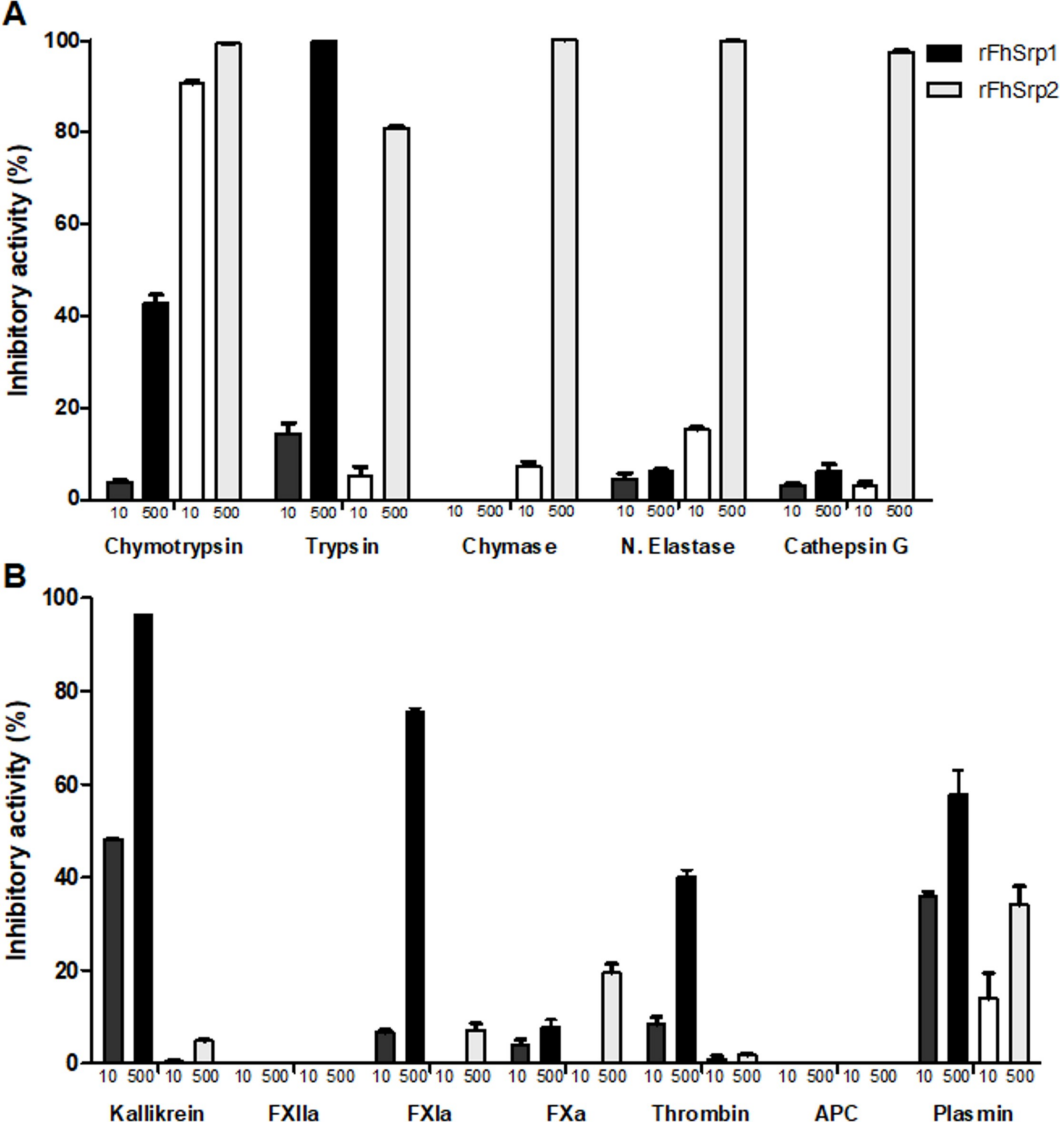
**Inhibitory activity of *F*. *hepatica* serpins against a panel of serine proteases involved in (A) digestion and host defence, and (B) coagulation**. The activity of chymotrypsin (4.0 nM), trypsin (168 nM), chymase (0.6 μM), neutrophil elastase (50 nM), cathepsin G (100 nM), kallikrein (150 nM), FXIIa (7.6 nM), FXIa (3.68 nM), FXa (5.8 nM), thrombin (800 pM), activated protein C (APC) (0.5 μM) and plasmin (800 μM) were tested in the presence of 10 and 500 nM rFhSrp1 (dark bars) or rFhSrp2 (light bars). Inhibition is presented relative to the activity of each enzyme in the absence of inhibitors and error bars indicate standard deviation of three separate experiments.

Throughout its migration in the mammalian host, *F*. *hepatica* encounters a range of host molecules. We have shown that *FhSrp1* and *FhSrp2* are transcribed in the intestinal, liver and bile ducts stages of the lifecycle stages within the mammalian host. Similarly, both serpins have also been identified in the secretomes of the NEJ and adult parasites [6, 14], highlighting that these inhibitors play an important role at the host-parasite interface throughout infection. Using a range of serine proteases involved in digestion, host defences and coagulation, we showed that rFhSrp1 and rFhSrp2 are functional inhibitors with distinct specificities (Fig 6).

**Fig 7:**
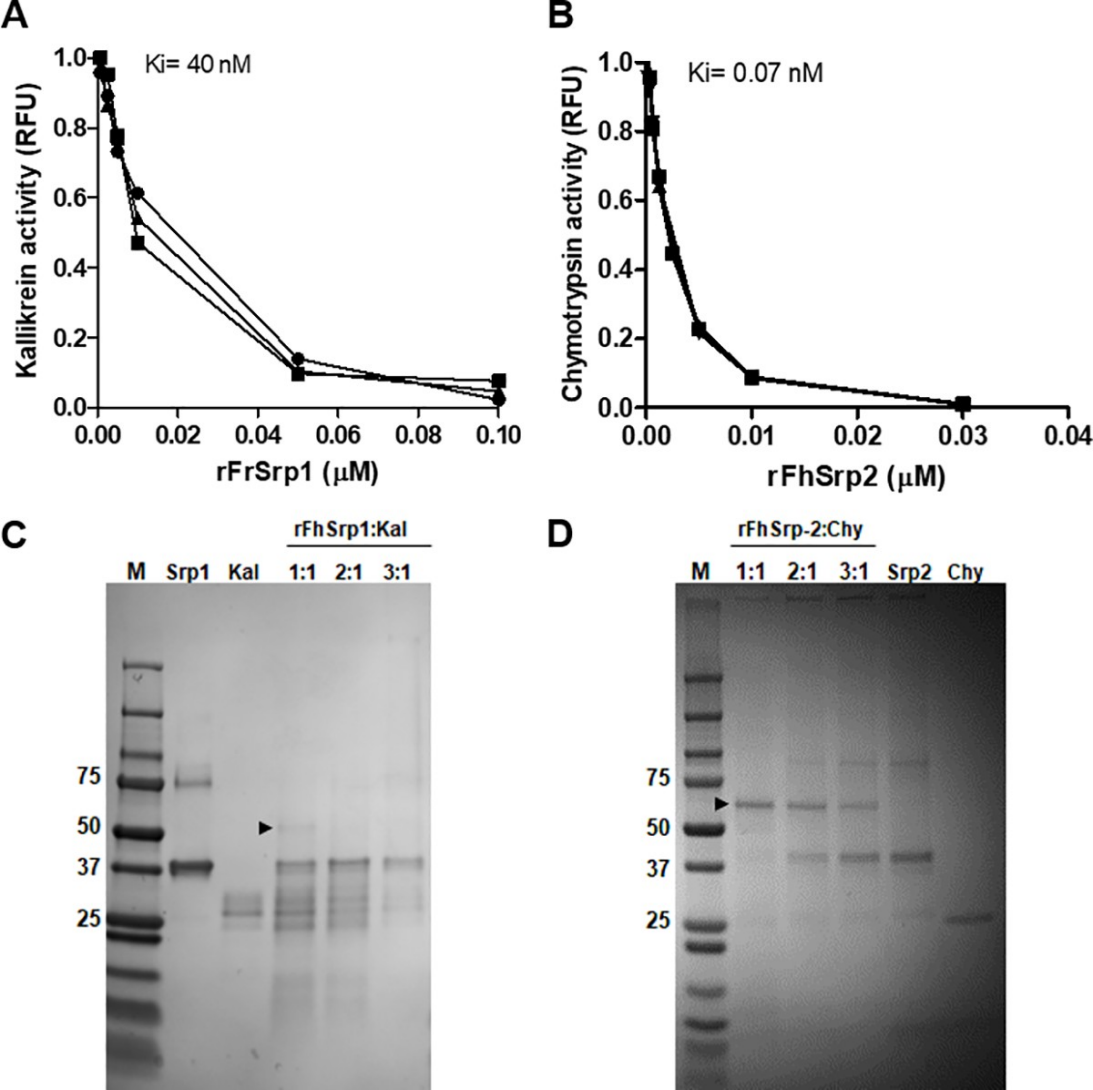
*hepatica* serpins inhibit specific serine proteases by forming covalent complexes. ***F*.** (A) Inhibition constant (*K*_i_) of kallikrein as a function of rFhSrp1 concentration. (B) Inhibition constant (*K*_i_) of chymotrypsin as a function of rFhSrp2 concentration. The curves represent the best fit to the Morrison equation (C) Serpin/serine protease complex formation between rFhSrp1 (Srp1) and kallikrein (Kal) was analyzed by SDS-PAGE under non-reducing conditions at serpin/protease ratios of 1:1, 2:1 and 3:1. Covalent complex formation occurs at ~55 kDa (black arrowhead) (D) Serpin/serine protease complex formation between rFhSrp2 (Srp2) and chymotrypsin (Chy) was analyzed by SDS-PAGE under non-reducing conditions at serpin/protease ratios of 1:1, 2:1 and 3:1. Covalent complex formation occurs at ~60 kDa (black arrowhead). Molecular weight (M) in kDa.

rFhSrp1 did not exhibit high potency against various serine proteases that we have classified generally as being involved in digestive and inflammatory functions (Fig 6A). The inhibitor did inactivate trypsin but only at the highest concentration examined (500 nM). rFhSrp1 was a more effective inhibitor of serine proteases involved in coagulation, namely kallikrein, FXIa, thrombin and plasmin (Fig 6B). Amongst these enzymes, rFhSrp1 was most effective against kallikrein. Specifically, at 10 and 500 nM, rFhSrp1 reduced kallikrein activity by 48% (±0.1) and 96% (±0.3), respectively (Fig 6B). The inhibitory constant, *K*_i_, for kallikrein was determined to be 40 nM (Fig 7A).

Conversely, rFhSrp2 was found to strongly inhibit chymotrypsin and chymotrypsin-like serine proteases involved in digestion and inflammation. At 500 nM, the activity of chymotrypsin-like proteases involved in inflammation, namely chymase, neutrophil elastase and cathepsin G, was reduced by 100% (±0.3), 99.9% (±0.01), and 97.5% (±0.4), respectively. In contrast, total inhibition of the activity of α-chymotrypsin was observed at 10 nM (Fig 6A). Kinetic analysis further supported that rFhSrp2 is a potent inhibitor of chymotrypsin. We determined that the inhibition constant, *K*_i_, is 0.07 nM (Fig 7B), which is significantly lower than that observed for the other chymotrypsin-like serine proteases used in this study, as their activity only slightly changed in the presence of 10 nM rFhSrp2 (Fig 6A).

The classical serpin inhibition mechanism involves the attack and cleavage of the scissile bond in the RCL by the target protease, resulting in the formation of a stable covalent linkage between protease and serpin that can be verified by gel electrophoresis [18]. In this study, we demonstrated the formation of the complex between serpin and target protease by incubating both, rFhSrp1-kallikrein or rFhSrp2-chymotrypsin, at various ratios for 30 min before boiling in SDS-PAGE sample buffer and analysis by SDS-PAGE. This analysis shows that co-incubation of rFhSrp1 and kallikrein did not result in a predominant stable complex and although a minor complex is formed (~55 kDa band) the majority is likely RCL-cleaved rFhSrp1 (Fig 7C, lanes 3 to 5). By contrast, the covalent complexing of rFhSrp2 with chymotrypsin resulted in the formation of a highly stable SDS complex of the expected size of ~60 kDa (Fig 7D, lanes 1 to 3), showing that in this case the inhibitory reaction is clearly favoured over the degradation of the inhibitor.

Using the activated partial thromboplastin time (APTT) test and the thrombin generation test, we showed that adding up to 50 μg/mL of rFhSrp1 or rFhSrp2 to the reaction does not alter coagulation (S4 Fig). These studies indicate that the recombinant *F*. *hepatica* rFhSrp1 or rFhSrp2 do not interfere with host clotting activity.

### Molecular homology modelling of FhSpr1-kallikrein and FhSpr2-chymotrypsin complexes

**Fig 8:**
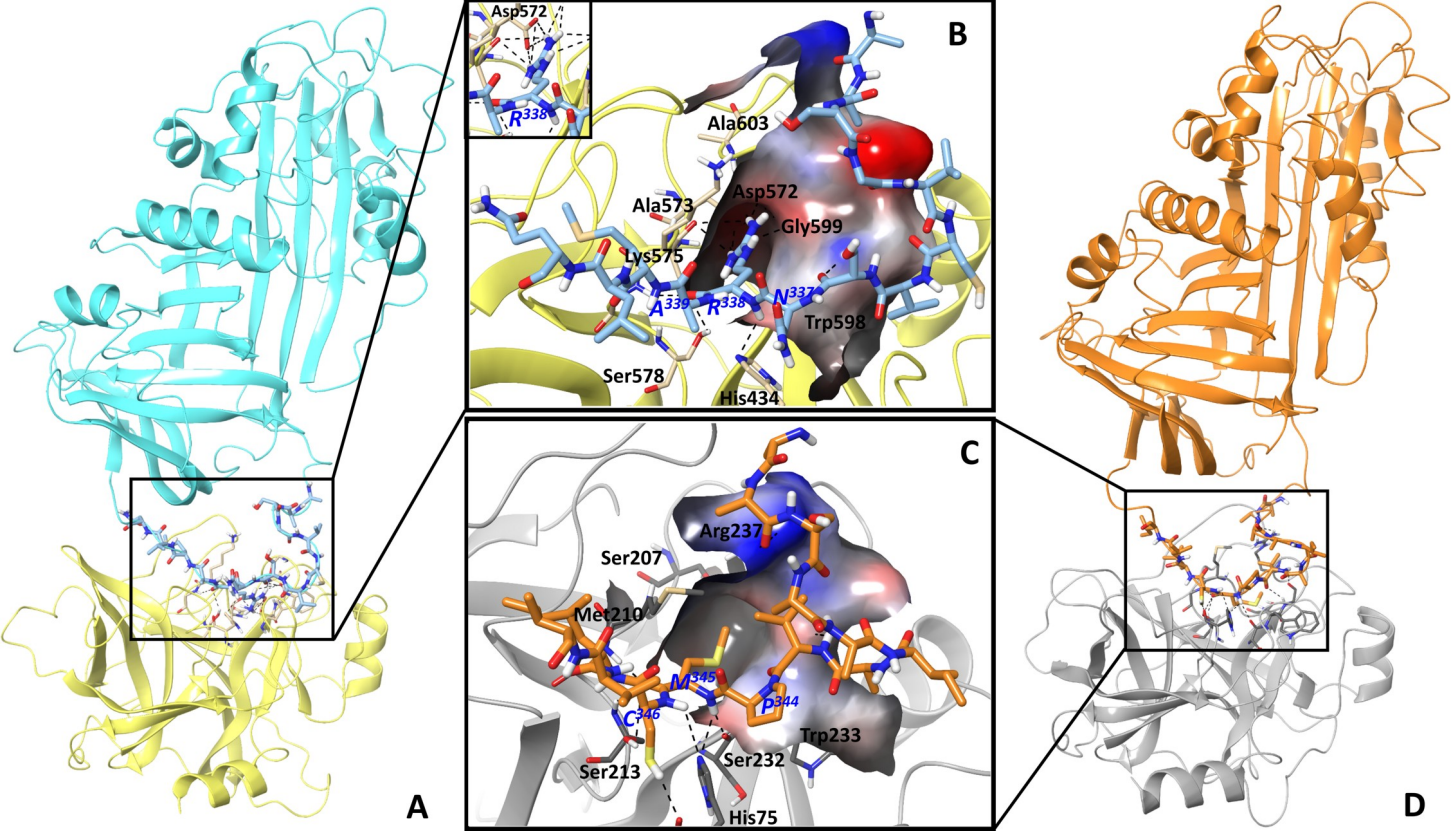
Molecular homology modelling of FhSpr1-kallikrein and FhSpr2-chymotrypsin complexes. The homology models of FhSpr1 and FhSpr2 were built based on *Manduca sexta* serpin 1B (PDB code: 1K9O), whereas the structures of kallikrein and chymotrypsin were rebuilt from the corresponding crystal structures of these enzymes with PDB codes, 1CBW and 5TJX, respectively. The protein-protein complexes were obtained via the protein structure alignment to the serpin-trypsin complex (PDB code: 1K9O). (A) Overall structure of the FhSpr1-kallikrein complex is shown in the cyan and yellow cartoons, respectively. (D) Overall structure of the FhSpr2-chymotrypsin complex is shown in the orange and grey cartoons, respectively. (B) and (C) Zoomed in views of the FhSpr1 and FhSrp2 serpin reactive centre loop (RCL) within the active site of the kallikrein and chymotrypsin serine proteases, respectively. The interacting residues of the serine proteases and the serpin RCL are visualized in stick-like representation. The P1 pocket and the linked area are shown as surface-like representations coloured with the electrostatic potential. Hydrogen bonds and a salt bridge are highlighted in black. Residue labels for kallikrein and chymotrypsin are shown in black and in blue for FhSpr1 and FhSpr2. In the FhSpr1-kallikrein complex (B) the R^338^ residue of FhSpr1 is within the narrow negatively changed S1 pocket that forms the salt bridge interaction with Asp^572^ and hydrogen bonds with the backbone of Gly^599^, Ala^603^ and Ala^573^. The polar interactions between the Arg^338^ and Asp^572^ residues are shown in the insertion image. In the FhSpr2-chymotrypsin complex (C), the M^345^ residue of FhSpr2 sits closely within the spacious uncharged S1 pocket forming many Van der Waals interactions with the residues of the pocket that collectively contribute to the overall tight binding.

We have explored the structural basis of FhSpr1 and FhSpr2 selectivity for specific serine proteases by constructing the complexes of FhSpr1-kallikrein and FhSpr2-chymotrypsin (Fig 8) using the serpin-trypsin crystal structure (PDB code: 1K9O) as a template. The model shows that the P1-residue Arg^338^ of FhSpr1 binds tightly to the S1 pocket of kallikrein by forming a salt bridge with Asp^572^ and hydrogen bonds with the backbone of Gly^599^, Ala^603^ and Ala^573^. While all the serine proteases examined in Fig 6B have a negative charged residue in the S1 pocket, and are theoretically capable of binding to FhSpr1, the differences in the observed specificity can be attributed to varied interactions with the non-conserved loops that line the active site. Indeed, the sequence identity between the tested serine proteases is relatively low (22–37%), indicating that other interactions would provide binding selectivity.

While the narrow and negatively charged S1 pocket of kallikrein preferentially accommodates the positively charged P1 Arg of FhSpr1, it is not suitable for the hydrophobic P1 Met of FhSpr2 due to the pocket charge and size and, thus, explains why FhSpr2 does not inhibit kallikrein. By contrast, the P1 Met of FhSpr2 sits favourably in the large and uncharged S1 pocket of chymotrypsin. However, the complex is not stabilised by interactions between the P1 Met^345^ and the S1 Ser^232^ but rather by the highly complementary fit that allows substantial Van der Waals interactions with a number of residues within the active pocket (Fig 8C). FhSrp1 does not make such a ‘snug’ fit into the chymotrypsin pockets and, hence, does not inactivate this enzyme.

### *F*. *hepatica* serpins cross regulate cysteine proteases

**Fig 9:**
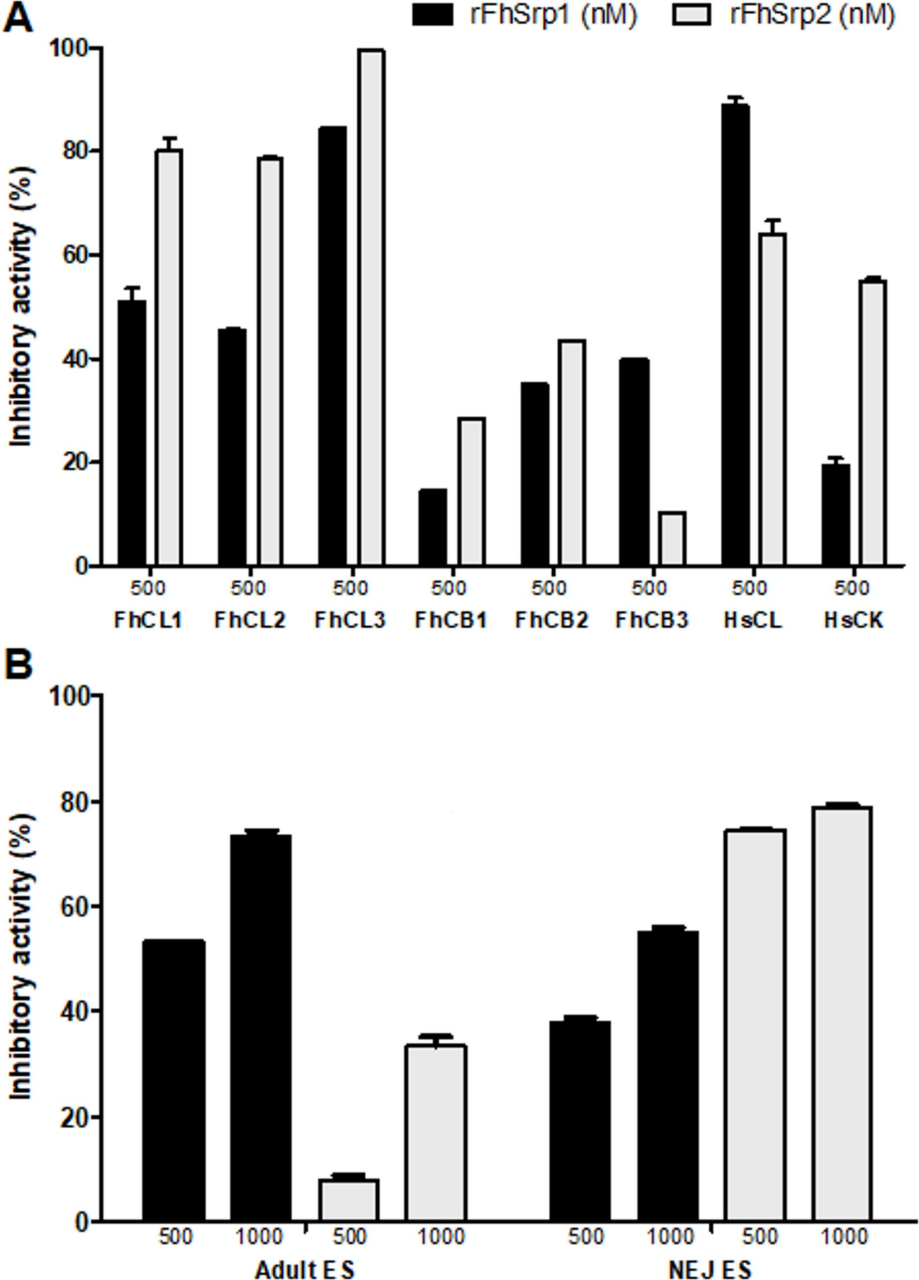
*F*. *hepatica* serpins inhibit parasite and host cysteine proteases. The activity of *F*. *hepatica* cathepsin cysteine proteases, FhCL1 (2.7 nM), FhCL2 (5.0 nM), FhCL3 (5.0 nM), FhCB1 (135 nM), FhCB2 (270 nM), FhCB3 (135 nM), and human cathepsin cysteine proteases, HsCL (0.2 nM) and HsCK (2.0 nM) were tested in the absence or presence of 500 nM rFhSrp1 (dark bars) or rFhSrp2 (light bars). (B) The activity of native *F*. *hepatica* cathepsin L proteases were assessed in the excreted/secreted proteins (ES) recovered from adult and NEJ 24 hr parasites in the presence of 500 and 1000 nM rFhSrp1 (dark bars) or rFhSrp2 (light bars). Inhibition is presented relative to the activity of each enzyme or extract in the absence of inhibitors and error bars indicate standard deviation of three separate experiments.

In addition to inhibiting serine proteases, studies have shown that serpins can also inhibit cysteine proteases [18]. In contrast to other helminths, *F*. *hepatica* relies almost exclusively on a family of expanded cathepsin L and B proteases to facilitate its migration and feeding within the mammalian host. We have previously shown that in addition to a range of cystatins, *F*. *hepatica* secretes a group of Kunitz-type serine proteases inhibitors that show an atypical adaptation to inhibit cathepsin L cysteine proteases [13]. We therefore investigated the inhibitory profile of the *F*. *hepatica* serpins against cathepsin proteases to determine if the expression of these inhibitors also represent further parasite-specific adaptations. While both rFhSrp1 and rFhSrp2 were found to inhibit recombinant cathepsin L proteases, namely FhCL1, FhCL2, FhCL3 (Fig 9A), this was observed only at the highest concentration tested (500 nM). Very low inhibitory activity against the cathepsin B proteases was detected. Both rFhSrp1 and rFhSrp2 (1 μM) inhibited the native cathepsin proteases found in the ES extracts of NEJ and adult parasites (Fig 9B). rFhSrp1 and rFhSrp2 also exhibit low levels of inhibition against the human cathepsin L and K proteases (Fig 9A).

### Recombinant *F*. *hepatica* serpins show efficacy as vaccine candidates

**Fig 10:**
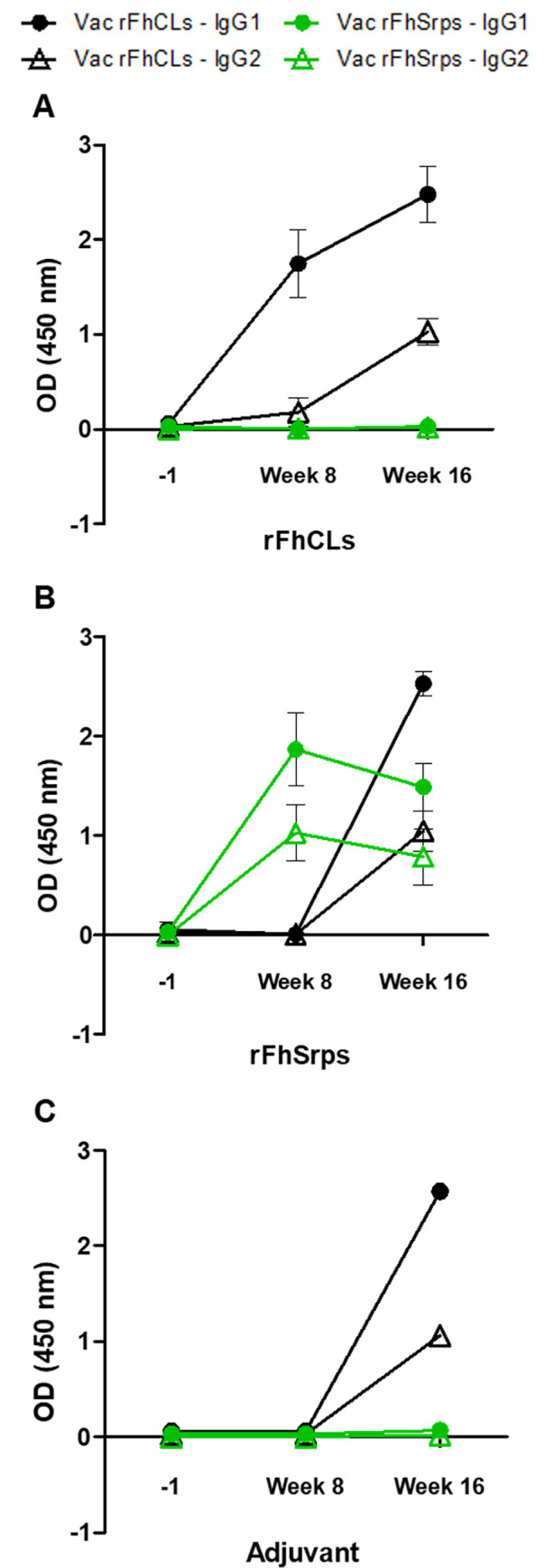
Antibody responses to the *F*. *hepatica* cathepsin proteases and serpin vaccine cocktails. Mean optical density values for IgG1 and IgG2 isotype antibodies to the recombinant cathepsin proteases (rFhCLs) and the recombinant serpins (rFhSrps) assessed within (A) the animals vaccinated with the rFhCLs, (B) the animals vaccinated with the rFhSrps and (C) in the adjuvant control group. Samples were assessed at day -1, week 8 and week 16. Error bars indicate standard deviation of three separate experiments.

Two independent trials were carried out to assess the efficacy of the recombinant serpins as vaccine targets to prevent fasciolosis in rats. As verified in the Montanide adjuvant control group, following challenge with *F*. *hepatica* metacercariae, the infected animals developed a natural immune response to FhCL1 dominated by an IgG1 isotype but did not elicit anti- rFhSrp1/rFhSrp2 responses showing that the serpins are not natural immunogens (Fig 10C).

**Fig 11:**
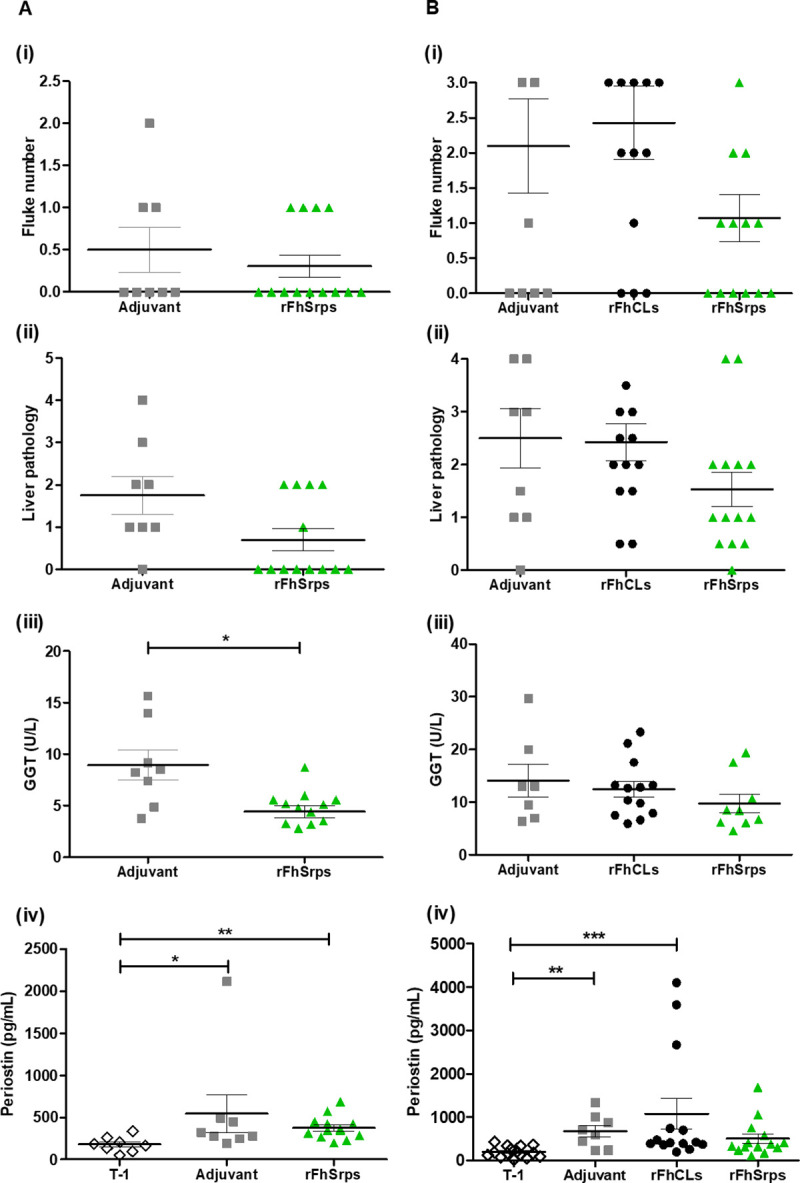
Evaluation of *F*. *hepatica* serpins as vaccine candidates. Results are presented for each trial individually, on the left the first trial (A) and on the right the second trial (B), representing the adjuvant control group (Adjuvant), the animals vaccinated with the *F*. *hepatica* recombinant cathepsin L2 and cathepsin L3 proteases (FhCLs) and the animals vaccinated with the *F*. *hepatica* recombinant serpin 1 and 2 (FhSrps). T-1 represents sera samples collected before vaccination at the beginning of the trial. (i) Number of flukes recovered from the bile duct; (ii) Liver pathology, (iii) Gamma-glutamyl transferase (GGT) sera levels and (iv) Periostin sera levels. Error bars indicate standard error of three separate experiments. Statistical differences between groups was assessed by Mann Whitney or One-way Anova followed by Kruskal-Wallis test (95% confidence intervals) (p<0.05: *; p<0.01: **; p<0.001: ***).

Analysis of the immune response of vaccinated animals demonstrated that an antibody response was generated to the recombinant rFhSrp1/rFhSrp2 within the serpin vaccine formulation in both trials (Fig 10, only data from trial 2 shown). At the time of challenge (week 8), both IgG1 and IgG2 isotype responses were elevated in the serpin vaccinated group. However, by week 16, eight weeks after challenge, the antibody levels to the serpins decreased. By contrast, challenge infection boosted anti-FhCL1 responses in both Montanide adjuvant controls and rFhCL2/rFhCL3-vaccinated animals (Fig 10A to 10C). Although not statistically significant, the rFhSrps vaccine group in both trials had lower numbers of parasites. They also showed lower levels of pathology based on clinical scores, and GGT and periostin serum levels (Fig 11A and 11B, panels ii, iii and iv).

GGT levels, used as an indirect marker of liver damage, were only detected in the sera at week 16 of trial 1 and were significantly lower in the rats vaccinated with rFhSrps (Fig 11A, panel iii). Our results show that periostin is consistently lower in rats vaccinated with the recombinant serpins when compared to rats in the adjuvant or rFhCLs vaccinated groups. Moreover, in the second trial that involved a larger number of animals, periostin levels in the group vaccinated with the recombinant serpins were statistically the same as that detected in control serum collected before vaccination (Fig 11B, panel iv). Periostin levels in serum were positively correlated with adult fluke number (0.3, p < 0.05), liver pathology (0.4, p < 0.005), and GGT levels (0.5, p < 0.005), indicating its suitability as a biomarker to assess liver pathology and vaccine efficacy in rodent trials.

## Discussion

Serpins are one-use suicide substrate inhibitors of serine and, albeit less commonly, cysteine proteinases [16]. They possess a reactive centre loop (RCL), which contains the recognition sequence for the active site of their target proteases. Following insertion of the RCL into the active site pocket, the inhibitory mechanism involves cleavage of the P1-P1’ bond that induces trapping of the protease-inhibitor into a stable covalent complex, which ultimately results in conformational distortion and inactivation of the protease [18, 42]. In this study, we show that *F*. *hepatica* transcribes a multi-gene family of serpins composed of seven members, *FhSrp1–7*. Several members are highly expressed by the NEJ and adult parasites (*FhSrp1–4*), whereas *FhSrp5*, *FhSrp6* and *FhSrp7* are constitutively expressed throughout the lifecycle and at lower transcription levels. The strict temporal developmental regulation of the various serpin family members is reminiscent of the multi-gene families of cathepsin L and cathepsin B proteases [11], the cystatin cysteine protease inhibitors [12] and the atypical Kunitz-type cysteine protease inhibitors [13] suggesting that *F*. *hepatica* possesses a plethora of inhibitors to regulate the protease/anti-protease balance throughout its migration, growth and development in the mammalian host.

The four serpins (FhSrp1-FhSrp4) that are transcribed in the infectious NEJ stage together represent 3% of the total protein secreted by the NEJ, which could be considered relatively low (cysteine proteases represent 80% of the secretome). What is more relevant, however, is that *F*. *hepatica* does not secrete serine proteases [6, 14] and, therefore, we speculate that the target for the serpin inhibitors are host serine proteases. We chose two serpins, FhSrp1 and FhSrp2, for recombinant expression and further characterisation as these are highly expressed serpins in the infectious NEJ. Consistent with their distinct RCL structures the two serpins exhibited different inhibitory profiles when screened against a broad panel of host serine and cysteine proteases. This screen revealed that rFhSrp1 potently inhibits kallikrein (*K*_i_ = 40 nM), but less strongly regulates the activity of other trypsin-like proteases including trypsin, FXIa, thrombin and plasmin. By marked contrast, rFhSrp2 was not effective against the trypsin-like proteases but was a very potent inhibitor of chymotrypsin (*K*_i_ = 0.07 nM) and other chymotrypsin-like proteases such chymase, cathepsin G and neutrophil elastase.

Molecular modelling using a previously determined serpin-protease crystal structure (PDB code: 1K9O) as a template helped explain the selective inhibitory activity of rFhSrp1 and rFhSrp2. For rFhSrp1, the P1 Arg residue of the scissile bond is the primary determinant of the selectivity for its cognate protease. However, since trypsin, plasmin, FXIa, thrombin and kallikrein all have a marked preference for a P1 Arg residue in their substrates [43, 44], the specificity of rFhSrp1 for kallikrein was attributed to it having an active site with the ‘best-fit’ for the RCL. Indeed, our *in silico* model of FhSrp1-kallikrein stressed the contribution of the residues Asp at P2 and Ser at P3 to the efficient binding and serpin-protease complex stabilization, together with interactions that take place between the serpin and non-conserved loops that lie at the rim of the protease active site. On the other hand, the specificity of rFhSrp2 for the chymotrypsin-like enzymes is governed not by the P1 residues but by many Van der Waals interactions that occur between the highly complementary RCL and the chymotrypsin active site, in addition to interactions with the non-conserved loop interactions. Gettins and Olson [45] showed that although 15 human serpins have an Arg or Lys residue at the P1 position each shows high inhibition specificity for their cognate proteases, indicating that adjacent residues and exosites are responsible for refining the serpin’s specificity. The impact of exosites on serpin activity was evident by the heparin-dependent inhibition of anti-thrombin towards thrombin and other proteases involved in coagulation [45]. In the present study, we found no significant improvement of the rFhSrp1 or rFhSrp2 inhibitory activity when heparan sulfate, low molecular weight heparin, dermatan or dextran sulfate were added to the reactions (S5 Fig).

Alignment of the RCL region from the seven *F*. *hepatica* serpins revealed that each family member has a conserved hinge region (P17 –P8) and shutter region, but exhibits a unique scissile P1-P1’ pair and surrounding sequence (P8 –P8’). This variance indicates that the genes encoding the inhibitors have evolved by duplication followed by divergence to create a family that could target a wide variety of proteases and play important roles modulating a range of host cellular and physiological functions during infection. Predicting the specificity of each of these inhibitors without 3-D structures is still a challenge, especially for parasite serpins that diverge greatly from human serpins in terms of RCL structure. Our broader phylogenetic studies of serpins in parasitic flatworms has revealed a great variety of serpin RCLs amongst these helminths that, by extension, suggests a broad target profile of serine proteases. Given that serine proteases represent over one-third of known invertebrate proteases and are involved in a range of biological functions including development, fertilisation, blood coagulation, fibrinolysis and immune responses [46] the precise targets for each of these serpins is speculative. Then again, this array of RCLs represents an opportunity to identify new inhibitors with important biological or biotherapeutic potential.

The high expression and secretion of serpins by NEJ indicates that they are essential in the early phase of the parasite’s invasion of the vertebrate host. Despite the fact that FhSrp1–4 were identified in the *F*. *hepatica* ES products none exhibit signal peptides which indicates that they are released into host tissues through an alternative pathway. Recently, de la Torre-Escudero et al. [47] identified FhSrp1-4 within *F*. *hepatica* secreted extracellular vesicles (EV) from where they likely are delivered into the host tissues at high concentration and/or into host immune cells such as macrophages and neutrophils. It is noteworthy that rFhSrp2 efficiently inhibits lysosomal serine proteases such cathepsin G and elastase, which are expressed predominantly in neutrophils and monocytes/macrophages [48] and play a central role during inflammatory responses by attracting monocytes [49]. Interestingly, a recombinant *Trichinella pseudospiralis* serpin with similar activity to rFhSrp2, *Tp*-Serpin, was demonstrated to induce alternative activated macrophages (M2 phenotype) *in vitro* [50]. It is possible that *F*. *hepatica* serpins are involved in the task of influencing host innate immune cells and contributing to the induction of Th2-polarised responses as the parasite crosses the intestine and makes its way to the liver.

Several studies have suggested that regulation of the digestive and immune properties of endogenous and host serine proteases by specific serpins assists parasites during invasion and allows them to survive in intradermal and intravenous environments [25, 31, 33]. Following excystment within the gut, NEJ would be particularly vulnerable to digestive serine proteases such as chymotrypsin and trypsin unless they produced potent inhibitors to counter-act these activities. The very potent inhibitory activity of rFhSrp2 towards chymotrypsin (*K*_*i*_ = 0.07 nM) suggests that it helps the parasite to survive while crossing the intestinal wall to infect the host. Like rFhSrp2, the majority of trematode native or recombinant serpins primarily or strictly inhibit chymotrypsin or chymotrypsin-like proteases with variable potency [24, 25, 31–33] implying that regulation of such enzymes is a conserved mechanism in these parasites. However, only two reported *Schistosome* serpins showed inhibition of their target protease with similar potency to rFhSrp2; these are SjB10 (SjSrp4 within S2 Fig) which inhibited pancreatic elastase when used at 2.5 nM [33] and SjSPI (SjSrp3 within S2 Fig) that abrogated 90% of the chymotrypsin activity at 15 nM [19].

As mentioned above, in vertebrates, serpins are crucial in the regulation of serine proteases involved in complex proteolytic cascades such as blood coagulation, fibrinolysis and complement activation [18]. The specific inhibition of kallikrein (*K*i = 40 nM) by rFhSrp1 suggests that *F*. *hepatica* manipulates these processes as similar inhibitory activity has not been reported for other trematodes. A serpin from the carp flatworm *Eudiplozoon nipponicum* (EnSerp1) inhibited 17% of kallikrein activity when used at 425 nM [51], and AgSRPN6, a serpin from the African malaria mosquito *Anopheles gambiae*, inhibited 75% of protease activity when used at a rAgSRPN6/kallikrein ratio of 10∶1 [52]. The potency of rFhSrp1 resembles the human serpin C1-inhibitor, the most important physiological inhibitor of plasma kallikrein and coagulation factors FXIa and FXIIa [53]. However, our studies showed that rFhSrp1 does not affect host clotting ability and, hence, rFhSrp1 may better be considered in the context of inflammation since one of the major functions of kallikrein is to cleave the high molecular weight kininogen (HMWK) to generate the pro-inflammatory bradykinin (BK), a small peptide that mediates vascular leakage and causes pain sensation [54]. By limiting the kallikrein activity, *F*. *hepatica* could indirectly reduce inflammation and pathology during infection.

Unlike other trematode serpins previously characterized, we show in this study that rFhSrp1 and rFhSrp2 also cross-inhibit native and recombinant cathepsin L cysteine proteases of *F*. *hepatica* and also cathepsin L and K from humans. However, significant inhibition was only observed when the inhibitors were examined at relatively high concentration, 500 nM. Considering that *F*. *hepatica* secretes several cystatins [12] and atypical Kunitz-like inhibitors [13] that are active against parasite and host cathepsin cysteine proteases in the nano-molar range (<1 nM) we do not consider the regulation of the activity of these enzymes a primary function of serpins, although we cannot rule this out.

Serine protease activity is inhibited when a covalent complex is formed with the serpin RCL. Complete inhibition is ensured when a conformational change in the serpin protein occurs upon cleavage at the P1-P1^’^ bond, allowing rapid insertion and covalent binding of the protease into the β-sheet A of the inhibitor. Therefore, the initial interaction between a serpin and a target protease can generate two reaction products, the covalent 1:1 serpin-protease complex or an inactive RCL-cleaved serpin [16]. In this study we did not observe a stable complex between rFhSrp1 and kallikrein such that a RCL-cleaved rFhSrp1 is the most dominant product of the reaction. By contrast, the FhSrp2 formed a stable covalent complex with chymotrypsin suggesting that complex formation may depend on the strength of the inhibitor-protease interaction. Covalent bonding, therefore, may be related to the strength of binding which was >500-fold less for the FhSrp1-kallikrien complex than for the FhSrp2-chymtrypsin complex.

Our phylogenetic analysis of the flatworm serpin sequences revealed that the RCL sequences from the cestodes *D*. *latus*, *H*. *microstoma* and *S*. *erinaceieuropaei* showed 100% sequence similarity to that of the FhSrp2 RCL, an unusual finding given the high variability within the phylum Platyhelminthes serpin RCL sequences. While each tapeworm differs in its preferred definitive host (fish, rodents, cats/dogs, respectively), their mature adult stages reside in the small intestine. It is almost certain that these cestode serpins have a similar inhibitory profile to the *F*. *hepatica* FhSrp2 and, therefore, are potent inhibitors of chymotrypsin and chymotrypsin-like proteases. Intriguingly, no other serpin sequence from the 17 Platyhelminthes genomes interrogated as part of this study showed such high levels of similarity to the *F*. *hepatica* sequences. Even two serpins, EcSrp1 (ECPE_0000939301) and EcSrp2 (ECPE_0001149301), obtained from *E*. *caproni*, which resides within the small intestine and is the closest relative to *F*. *hepatica*, did not share the same level of sequence similarity to FhSrp2. Both EcSrp1 and EcSrp2 have been reported in the ES products of adult *E*. *caproni* parasites [55], indicating their utility within the intestinal niche. The convergence of the cestode sequences with that of *F*. *hepatica* FhSrp2 remains a curiosity at present but may add weight to the idea that inhibition of digestive serine proteases, particularly chymotrypsin, by serpins is important for intestinal parasites.

The emergence of drug-resistant parasites [56] hastens the need for new methods of control for fasciolosis, and vaccines are considered the more practical, environmental friendly and sustainable means of doing this. Inducing protective responses using molecules that are essential to parasite-host interactions in formulations, specifically those expressed by NEJ, is a rational approach to generating a potent long-term humoral and cellular immune response and prevent any pathology associated with the infection [57]. Trematode serpins are relevant in this context as well as having some precedent in other parasites. For example, Yan et al. [58] obtained a 36% reduction in challenge worm burden in mice by vaccinating with a recombinant *S*. *japonicum* serpin. Similarly, a vaccine that combined two *Clonorchis sinensis* serpins, rCsproSERPIN and rCsSERPIN2, induced strong IFN-γ production and IgG2a levels higher than IgG1 in rats, highlighting the immunoregulatory role of these inhibitors [32].

In this study, we performed two independent vaccine trials and compared two vaccine formulations containing (1) a combination of rFhSrp1 and rFhSrp2 and (2) a combination of recombinant *F*. *hepatica* cathepsin L2 (rFhCL2) and L3 (rFhCL3) formulated in the mineral-oil based adjuvant Montanide ISA 206VG. The studies were performed in rats as these exhibit a high level of natural resistance to the acute effects of primary and secondary infections [59]. Surprisingly, we observed that rats infected with *F*. *hepatica* do not develop antibodies to rFhSrp1/2 although a humoral antibody response was induced by vaccination. Complementary isotype analysis showed higher levels of IgG2 antibodies to rFhSrp1/2 than to rFhCLs, suggesting that serpins can stimulate a mixed Th1/Th2 response in rats more efficiently than the cathepsin-based vaccine. However, this specific response was not boosted by challenge infection and a correlation between parasite burden or tissue pathology and antibody levels was not observed.

As indicated by parasite enumeration and liver pathology, the rFhCLs vaccine elicited no significant protection and, thus, our results diverge from those obtained by vaccinating cattle or sheep with *F*. *hepatica* cathepsins, which induced protection levels ranging from 38 to 83% depending on the vaccine formulation used [60–62]. Jayaraj et al. [62] observed 83% reduction in fluke burden in rats vaccinated with recombinant *F*. *hepatica* cathepsins expressed by NEJ with Quil A as adjuvant. The rFhSrp1/2-vaccinated group yielded fewer liver flukes than the adjuvant control or the rFhCLs vaccinated animals but did not show significance. The vaccine resulted in a reduction in mean liver pathology assessed by visual evaluation and by detection of GGT and periostin in sera. High levels of serum periostin have been associated with poor prognosis of different liver diseases, including hepatic steatosis, inflammation and fibrosis [63]. In the fasciolosis context, we previously identified periostin as a molecular marker of parasite-induced liver pathogenesis in sheep [64] and here we demonstrate that serum periostin levels agree with fluke number, liver pathology and GGT levels indicating its suitability as a measure of liver pathology during chronic liver fluke infections. Although the rFhSrp1/2 vaccine data did not show a significant level of protection, the vaccine performed better than the rFhCLs that have been reported by various groups to be protective against *F*. *hepatica* [60, 62]. Therefore, considering the positive features observed with the rFhSrps vaccine in rodents, including its formulation in a commercially approved adjuvant, Montanide ISA 206VG, our data encourages more extensive vaccines trials, perhaps in combination with other antigens, in ruminants.

Until now, our knowledge of serpin inhibitors expressed by *F*. *hepatica* was limited. This work highlights the complexity and diversity of the liver fluke serpin family and sheds new light on the molecular interaction between parasite and host. The distinct and profound inhibitory activities of rFhSrp1 and rFhSrp2 reinforces that *F*. *hepatica* expresses serpins to regulate a range of proteolytic and inflammatory host responses to facilitate parasite survival during infection. A fuller understanding of the role of serpins during *F*. *hepatica* infections will come from the characterization of the remaining serpin members and vaccine trials designed to disrupt their functions.

## Supporting information

S1 Fig Alignment of the seven *F*. *hepatica* serpin RCL region taken from the MAFFT alignment used to generate the phylogenetic tree. Shading in grey represents similarity between the protein sequences. The P1 position is highlighted in yellow.
(DOCX)

S2 Fig Phylogenetic analysis of the relatedness of serpins within the phylum Platyhelminthes. (A) Midpoint-rooted maximum likelihood phylogram based on the protein sequence Arg^224^-Glu^374^ (FhSrp1 nomenclature) that includes the reactive centre loop (RCL) region, from representative serpin protein sequences from 18 helminth species: *Clonorchis sinensis* (CsSrp1-CsSrp4), *Echinococcus granulosus* (EgSrp1), *E*. *multilocularis* (EmSrp1), *Echinostoma caproni* (EcSrp1-EcSrp2), *Fasciola hepatica* (FhSrp1-FhSrp7), *Hymenolepis diminuta* (HdSrp1), *H*. *microstoma* (HmSrp1), *H*. *nana* (HnSrp1), *Macrostomum lignano* (MlSrp1), *Opisthorchis felineus* (OfSrp1-OfSrp4), *O*. *viverrini* (OvSrp1-OvSrp4), *Paragonimus westermani* (PwSrp1), *Schistocephalus solidus* (SsSrp1), *Schistosoma haematobium* (ShSrp1-ShSrp5), *S*. *japonicum* (SjSrp1-SjSrp4), *S*. *mansoni* (SmSrp1-SmSrp6), *S*. *margrebowiei* (SmrSrp1), and *S*. *rodhaini* (SrSrp1). The three clusters are indicated by the letters on the right. Bootstrap support values (1000 iterations) are shown at each node. Accession number/protein identifiers used for the phylogenetic analysis are presented in S2 Table. (B) Alignment of the serpin RCL region taken from the MAFFT alignment used to generate the phylogenetic tree. Shading in grey represents similarity between the sequences. The P1 position is highlighted in yellow.
(DOCX)

S3 Fig Amino acid ClustalW alignment comparing FhSrp2 with three cestode serpins. Serpin sequences from *Dibothriocephalus latus* (DILT_0001377201), *Hymenolepis microstoma* (HmN_003003780) and *Spirometra erinaceieuropaei* (SPER_0003298501) show 100% amino acid similarity to FhSrp2 across the entire length of the serpin sequence.
(DOCX)

S4 Fig Activated partial thromboplastin time (APTT) and the thrombin generation in the presence of *F*. *hepatica* recombinant serpins. (A) The inhibitory potential of the *F*. *hepatica* serpins against the intrinsic coagulation pathway was investigated using the APTT test performed in triplicate, in the presence of rFhSrp1 or rFhSrp2 at 50 μg/mL. Control, plasma without the recombinant *F*. *hepatica* serpins. (B) Thrombin generation was tested in triplicate in the presence or absence of rFhSrp1 (Bi) and rFhSrp2 (Bii), which were added in varying concentrations (12.5 to 50 μg/mL). Control, plasma without the recombinant *F*. *hepatica* serpins. Sec, seconds.
(DOCX)

S5 Fig Influence of glycosaminoglycans in the inhibitory activity of *F*. *hepatica* serpin against a panel of serine proteases. The activity of chymotrypsin (4.0 nM), trypsin (168 nM), neutrophil elastase (50 nM), cathepsin G (100 nM), kallikrein (150 nM), thrombin (800 pM) and plasmin (800 μM) were tested in the presence of 10 nM rFhSrp1 alone (dark bars) or in association with dextran sulfate (DS) (10 ng/mL) (dark grey bars) or in the presence of 10 nM rFhSrp2 alone (white bars) or in association to DS (10 ng/mL) (light grey bars). Inhibition is presented relative to the activity of each enzyme in the absence of inhibitors and error bars indicate standard deviation of three separate experiments.
(DOCX)

S1 Table Gross pathology assessment score for hepatic damage following *F*. *hepatica* infection. (DOCX)

S2 Table Accession number/protein identifiers of the sequences used for the phylogenetic analysis. (DOCX)
