# Supplementary material for: Fasciola hepatica serine protease inhibitor family (serpins): Purposely crafted for regulating host proteases
Source: PLoS Negl Trop Dis. 2020 Aug 6;14(8):e0008510. doi: 10.1371/journal.pntd.0008510 (PMC7437470; doi:10.1371/journal.pntd.0008510)
Supplement: S1 Fig — Shading in grey represents similarity between the protein sequences. The P1 position is highlighted in yellow. (DOCX) [file pntd.0008510.s001.docx]

P1

FhSrp1 EAGAVASAASGVCVS-NRAMLQP-IEFCADHAF

FhSrp3 EAGAVASAASGVCVS-NRSMLQP-IEFCADHAF

FhSrp2 EEGAEAAAASAAIAV-PMCLVIPEIQVKADHPF

FhSrp4 EGGAEAAAASAAIVRHGCCLAIPETQVKADHPF

FhSrp5 EIGVEAAAATSAVAV-PTSILNPTAEFHVDQPF

FhSrp6 EAGVEATAATAMMAV-PMSLLVPNVQFHVDQPF

FhSrp7 DLGITNQAVIASRKM------QAIREFRADHPF

**S1 Fig**
