# Supplementary material for: Fasciola hepatica serine protease inhibitor family (serpins): Purposely crafted for regulating host proteases
Source: PLoS Negl Trop Dis. 2020 Aug 6;14(8):e0008510. doi: 10.1371/journal.pntd.0008510 (PMC7437470; doi:10.1371/journal.pntd.0008510)
Supplement: S2 Fig — (A) Midpoint-rooted maximum likelihood phylogram based on the protein sequence Arg224-Glu374 (FhSrp1 nomenclature) that includes the reactive centre loop (RCL) region, from representative serpin protein sequences from 18 helminth species: Clonorchis sinensis (CsSrp1-CsSrp4), Echinococcus granulosus (EgSrp1), E. multilocularis (EmSrp1), Echinostoma caproni (EcSrp1-EcSrp2), Fasciola hepatica (FhSrp1-FhSrp7), Hymenolepis diminuta (HdSrp1), H. microstoma (HmSrp1), H. nana (HnSrp1), Macrostomum lignano (MlSrp1), Opisthorchis felineus (OfSrp1-OfSrp4), O. viverrini (OvSrp1-OvSrp4), Paragonimus westermani (PwSrp1), Schistocephalus solidus (SsSrp1), Schistosoma haematobium (ShSrp1-ShSrp5), S. japonicum (SjSrp1-SjSrp4), S. mansoni (SmSrp1-SmSrp6), S. margrebowiei (SmrSrp1), and S. rodhaini (SrSrp1). The three clusters are indicated by the letters on the right. Bootstrap support values (1000 iterations) are shown at each node. Accession number/protein identifiers used for the phylogenetic analysis are presented in S2 Table. (B) Alignment of the serpin RCL region taken from the MAFFT alignment used to generate the phylogenetic tree. Shading in grey represents similarity between the sequences. The P1 position is highlighted in yellow. (DOCX) [file pntd.0008510.s002.docx]

**A**

**
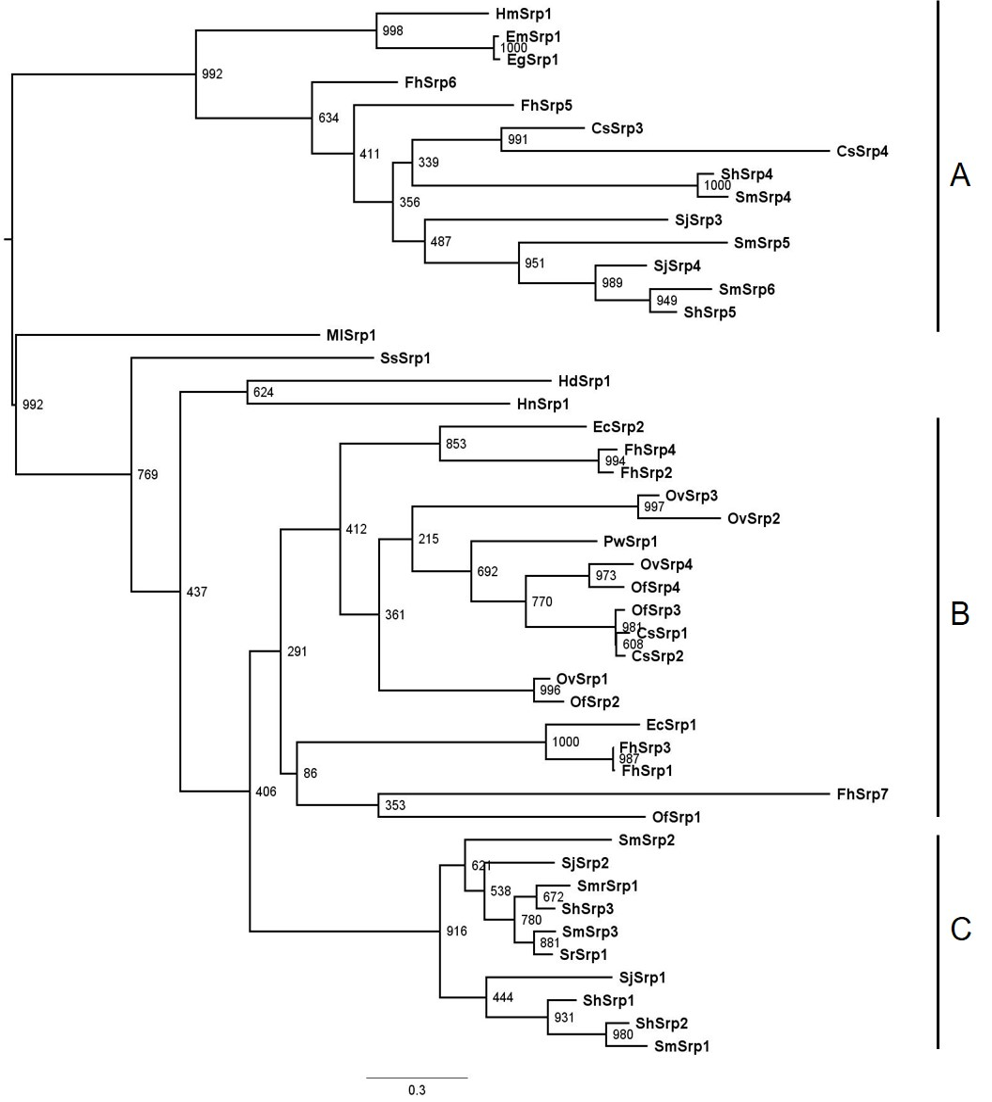
**

**B**

HmSrp1 EAGVKAAAVSGLNIM-PMSI--PP---PATPFHVDQPF

EmSrp1 EAGVKAAAVSGIEIL-PMSL--PP---PSVPFIVDQPF

EgSrp1 EAGVKAAAVSSIEIL-PMSL--PP---PSVPFIVDQPF

FhSrp6 EAGVEATAATAMMAV-PMSL--LV---PNVQFHVDQPF

FhSrp5 EIGVEAAAATSAVAV-PTSI--LN---PTAEFHVDQPF

CsSrp3 EAGVEAVSAVVGLAV-PMSL--VR---PEVMFHVNSTF

CsSrp4 ESGINIDMAQEIETA-STTE--GQ---TEVEVTANTTF

ShSrp4 EDGVEAAAATVMGIGFRSAR--PP---PSVRFDVNESF

SmSrp4 EDGVEAAAATVMGIGLRSAR--PP---PSIRFDVNESF

SjSrp3 EKGVEAAAATAIYSL-GRSLHYVP---TNAYFIADHPF

SmSrp5 EVGMEARSVANAMFI-PLSS--YR---NPIQFHITHPF

SjSrp4 ESGIEAASVTSPIIV-PISA--LI---PDVNFHVTHPF

SmSrp6 ESGIEATTVTSPIFV-PISA--VL---PDIDFNVNHPF

ShSrp5 ESGIEATTVTSPIFV-PFSA--II---PEVDFHVTHPF

MlSrp1 EEGAEAAAATAVAIA-CFSM--PM----MMPFFVTEPF

SsSrp1 EDGAEAAAATGISEGIECSM--PP---PPVDFKVTHPF

HdSrp1 EEGAEAAAATGMIMM-MRCM--PM---PPPNFLIDHPF

HnSrp1 EEGAEAAAATGMSIM-PMSL--------VPCIAADHPF

EcSrp2 EEGATAAATTYAIMT-NCYR--PPSP-PPIIVRVDHPF

FhSrp4 EGGAEAAAASAAIVRHGCCL--AI---PETQVKADHPF

FhSrp2 EEGAEAAAASAAIAV-PMCL--VI---PEIQVKADHPF

OvSrp3 EEGVTAAAATLFTVC-ASFQ--L-----LQTISVDHPF

OvSrp2 EEGVTAAAATLFTQS-FSTR--L-----PETISVDHPF

PwSrp1 EEGATAAAATAVMMNMRCAM--M-----RPTVRVDRPF

OvSrp4 EEGATAAAATGMVAN-YCSL--QV----NPPFVVDHPF

OfSrp4 EEGATAVAATAMMIN-RCAA--RR----PLSFVVDHPF

OfSrp3 EEGATAAASTGMMMC-RMMM--G-----PPPFRVDHPF

CsSrp1 EEGATAAASTGMMVA-RMMM--G-----PPPFRVDHPF

CsSrp2 EEGATAAASTGMMVS-RMMM--G-----PPPFRVDHPF

OvSrp1 EEGATAAAATGMVAV-AMCY--M----PTPVIQVDHPF

OfSrp2 EQGATAAAATGIMAV-PMCF--M----PKPVIQVDHPF

EcSrp1 EAGAVATGATGVGIA-NRSL--LR----PIVFNANHAF

FhSrp3 EAGAVASAASGVCVS-NRSM--LQ----PIEFCADHAF

FhSrp1 EAGAVASAASGVCVS-NRAM--LQ----PIEFCADHAF

FhSrp7 DLGITNQAVIASRKM-----------QAIREFRADHPF

OfSrp1 EGGVEAAAATGFEFA-FLSL--AK----PKKFRVDHPF

SmSrp2 EEGVVAAGVTACVFD-NCDS--ESSE-AEVEFRVDHSF

SjSrp2 EEGAVAASASATVMY-MCSA--IRSHQPVPEFRIDHPF

SmrSrp1 EKGAVAAAATAVEVT-NRSF--VRPHKPVPEFRVDHPF

ShSrp3 EKGAVAAAATATQMV-YCTS--LRPHKPVPEFRVDHPF

SmSrp3 EKGAVAAAATATRMI-RCTA--FIFHKPVPEFRVNHPF

SrSrp1 EKGAVAAAATAIQMV-RYSA--GRSYKHIPEFRINHPF

SjSrp1 EKGAEAAAATATKII-PLSL--CIDDEPLIEFRINHSF

ShSrp1 ERGAVAAAATSVEFI-NLSL--CESEEPEVEFRVDHPF

ShSrp2 EQGVVAAAASSAEVV-QLAA--PLPEFADEEFRVDHPF

SmSrp1 EQGVVAAAASSVEVV-QLSA--PLPEFSDEEFRVNHSF

**S2 Fig**
