# Supplementary material for: Fasciola hepatica serine protease inhibitor family (serpins): Purposely crafted for regulating host proteases
Source: PLoS Negl Trop Dis. 2020 Aug 6;14(8):e0008510. doi: 10.1371/journal.pntd.0008510 (PMC7437470; doi:10.1371/journal.pntd.0008510)
Supplement: S1 Table — (DOCX) [file pntd.0008510.s006.docx]

**S1 Table. Gross pathology assessment score for hepatic damage following *F. hepatica* infection.**

| **Score** | **Pathology** |
| --- | --- |
| 0 | No visible signs of pathology, including lesions and fibrosis and no visible parasites within the bile ducts. |
| 1 | Livers show swollen bile ducts typical of liver fluke infection, but no visible parasites |
| 2 | Mild damage, including localized lesions. |
| 3 | Moderate damage, with localized fibrosis. |
| 4 | Moderate to large areas of tissue of damage, localized fibrosis and focal abscesses. |
| 5 | Large areas of tissue with visible damage including fibrosis, focal abscesses and calcified bile ducts. |
