# Supplementary material for: Fasciola hepatica serine protease inhibitor family (serpins): Purposely crafted for regulating host proteases
Source: PLoS Negl Trop Dis. 2020 Aug 6;14(8):e0008510. doi: 10.1371/journal.pntd.0008510 (PMC7437470; doi:10.1371/journal.pntd.0008510)
Supplement: S2 Table — (DOCX) [file pntd.0008510.s007.docx]

**S2 Table. Accession number/protein identifiers of the sequences used for the phylogenetic analysis.**

| **Nomenclature on Phylogram** | **Accession number/WormBase ParaSite Identifier*** |
| --- | --- |
| *Clonorchis sinensis* |  |
| CsSrp1 | GAA28469 / Cs-k2.gene2069 |
| CsSrp2 | ADI60059 |
| CsSrp3 | GAA48350 / Cs-k2.gene8414 |
| CsSrp4 | GAA37554 / Cs-k2.gene8412 |
| *Echinococcus granulosus* |  |
| EgSrp1 | EUB63679 / EGR_01302 |
| *Echinococcus* *multilocularis* |  |
| EmSrp1 | CDS40644 / EmuJ_000824000 |
| *Echinostoma caproni* |  |
| EcSrp1 | ECPE_0000939301 |
| EcSrp2 | ECPE_0001149301 |
| *Fasciola hepatica* |  |
| FhSrp1 | MT419773 / BN1106_s3864B000104 /  maker-scaffold10x_114_pilon-snap-gene-0.90 |
| FhSrp2 | MT419774 / BN1106_s4618B000050 /  maker-scaffold10x_113_pilon-augustus-gene-0.45 |
| FhSrp3 | BN1106_s3226B000049 |
| FhSrp4 | BN1106_s1727B000096 /  maker-scaffold10x_794_pilon-snap-gene-0.129 |
| FhSrp5 | BN1106_s4565B000032 /  maker-scaffold10x_293_pilon-augustus-gene-0.19 |
| FhSrp6 | BN1106_s4565B000033 / BN1106_s284B000286  maker-scaffold10x_293_pilon-snap-gene-0.149  maker-scaffold10x_293_pilon-snap-gene-0.148 |
| FhSrp7 | BN1106_s554B000503 /  maker-scaffold10x_2113_pilon-snap-gene-0.16 |
| *Hymenolepis diminuta* |  |
| HdSrp1 | HDID_0000409001 |
| *Hymenolepis microstoma* |  |
| HmSrp1 | CDS32008 |
| *Hymenolepis nana* |  |
| HnSrp1 | HNAJ_0000265801 |
| *Macrostomum lignano* |  |
| MlSrp1 | BOX15_Mlig023593g2 |
| *Opisthorchis felineus* |  |
| OfSrp1 | CRM22_007225 |
| OfSrp2 | CRM22_010060 |
| OfSrp3 | CRM22_006113 |
| OfSrp4 | CRM22_006112 |
| *Opisthorchis viverrini* |  |
| OvSrp1 | T265_08625 |
| OvSrp2 | T265_10634 |
| OvSrp3 | T265_14285 |
| OvSrp4 | T265_11935 |
| *Paragonimus westermani* |  |
| PwSrp1 | ABV57466 |
| *Schistocephalus solidus* |  |
| SsSrp1 | SSLN_0001468201 |
| *Schistosoma haematobium* |  |
| ShSrp1 | KGB37771 / MS3_06131 |
| ShSrp2 | KGB37772 / MS3_06125 |
| ShSrp3 | KGB42280 / MS3_10931 |
| ShSrp4 | MS3_00995 |
| ShSrp5 | AAA19730 |
| *Schistosoma japonicum* |  |
| SjSrp1 | AAW25282 / Sjp_0113080 |
| SjSrp2 | Sjp_0076600 |
| SjSrp3 | Sjp_0113720 |
| SjSrp4 | AAK57435 |
| *Schistosoma mansoni* |  |
| SmSrp1 | Smp_062080 |
| SmSrp2 | Smp_155530 |
| SmSrp3 | Smp_305550 (Smp_155550) |
| SmSrp4 | CCD74817 / Smp_003300 |
| SmSrp5 | CCD60070 / Smp_090090 |
| SmSrp6 | Smp_090080 |
| *Schistosoma margrebowiei* |  |
| SmrSrp1 | SMRZ_0002479701 |
| *Schistosoma rodhaini* |  |
| SrSrp1 | SROB_0002079901 |

* WormBase ParaSite data (WBPS14) available at https://parasite.wormbase.org/index.html
